# Comparative DNA methylome analysis of endometrial carcinoma reveals complex and distinct deregulation of cancer promoters and enhancers

**DOI:** 10.1186/1471-2164-15-868

**Published:** 2014-10-06

**Authors:** Bo Zhang, XiaoYun Xing, Jing Li, Rebecca F Lowdon, Yan Zhou, Nan Lin, Baoxue Zhang, Vasavi Sundaram, Katherine B Chiappinelli, Ian S Hagemann, David G Mutch, Paul J Goodfellow, Ting Wang

**Affiliations:** Department of Genetics, Center for Genome Sciences and Systems Biology, Washington University School of Medicine, St. Louis, MO 63108 USA; Shanghai International Joint Cancer Institute, The Second Military Medical University, Shanghai, 200433 P. R. China; Key Laboratory for Applied Statistics of MOE, School of Mathematics and Statistics, Northeast Normal University, Changchun, Jilin Province 130024 P. R. China; Department of Mathematics and Division of Biostatistics, Washington University in Saint Louis, Saint Louis, MO 63130 USA; Department of Oncology, Sidney Kimmel Cancer Center, Johns Hopkins University, Baltimore, MD 21231 USA; Department of Pathology and Immunology, Washington University School of Medicine, St. Louis, MO 63110 USA; Division of Gynecologic Oncology, Department of Obstetrics and Gynecology, Washington University School of Medicine, St Louis, MO 63124 USA; The Ohio State University Comprehensive Cancer Center, The Ohio State University, Columbus, OH 43210 USA

## Abstract

**Background:**

Aberrant DNA methylation is a hallmark of many cancers. Classically there are two types of endometrial cancer, endometrioid adenocarcinoma (EAC), or Type I, and uterine papillary serous carcinoma (UPSC), or Type II. However, the whole genome DNA methylation changes in these two classical types of endometrial cancer is still unknown.

**Results:**

Here we described complete genome-wide DNA methylome maps of EAC, UPSC, and normal endometrium by applying a combined strategy of methylated DNA immunoprecipitation sequencing (MeDIP-seq) and methylation-sensitive restriction enzyme digestion sequencing (MRE-seq). We discovered distinct genome-wide DNA methylation patterns in EAC and UPSC: 27,009 and 15,676 recurrent differentially methylated regions (DMRs) were identified respectively, compared with normal endometrium. Over 80% of DMRs were in intergenic and intronic regions. The majority of these DMRs were not interrogated on the commonly used Infinium 450K array platform. Large-scale demethylation of chromosome X was detected in UPSC, accompanied by decreased *XIST* expression. Importantly, we discovered that the majority of the DMRs harbored promoter or enhancer functions and are specifically associated with genes related to uterine development and disease. Among these, abnormal methylation of transposable elements (TEs) may provide a novel mechanism to deregulate normal endometrium-specific enhancers derived from specific TEs.

**Conclusions:**

DNA methylation changes are an important signature of endometrial cancer and regulate gene expression by affecting not only proximal promoters but also distal enhancers.

**Electronic supplementary material:**

The online version of this article (doi:10.1186/1471-2164-15-868) contains supplementary material, which is available to authorized users.

## Background

Endometrial cancer is the most common gynecologic malignancy in the United States, with an estimated 47,130 new cases and 8,010 deaths annually [1]. Most cases of endometrial cancer are endometrioid adenocarcinoma (EAC), are of low grade, and are diagnosed at an early stage, with a 5-year survival rate of greater than 85% [1]. Uterine papillary serous carcinoma (UPSC), an aggressive histologic subtype of endometrial cancer, represents less than 10% of all endometrial cancers. However, UPSC accounts for more than 50% of recurrences and deaths attributed to endometrial carcinoma [2, 3]. EAC commonly displays near-diploid karyotypes, microsatellite instability, and mutations in the *PTEN*, *KRAS*, and *CTNNB1* (β-catenin) genes. UPSC is characterized by frequent *TP53* mutation, *Her-2*/*neu* overexpression, and an aneuploid karyotype [2, 3].

Like many malignancies, endometrial cancer is a complex disease driven by both genetic, epigenetic and environmental factors. DNA methylation has long been implicated in the development and progression of tumors in various tissue types [4–6]. An overall reduction in total 5-methylcytosine level and focal hypermethylation in CpG islands near tumor-suppressor gene transcriptional start sites were found in many different types of cancers [7–10]. Among endometrial cancers, promoters of important tumor suppressor genes including *MLH1*, *RASSF1A*, *PTEN*, and *APC*, were found to be hypermethylated in EAC. However, methylation status of these genes was largely unaltered in UPSC [11–16]. The divergent hypermethylation between these tumor types might be caused by the significantly increased expression of DNA methyltransferases *DNMT1* and *DNMT3B* observed in EAC, whereas expressions of these enzymes were unchanged or even decreased in UPSC [14, 17].

Recently, The Cancer Genome Atlas Consortium (TCGA) profiled DNA methylation of more than 300 endometrial cancer samples using array-based DNA methylation platforms (HumanMethylation27 BeadChip and HumanMethylation450 BeadChip), which interrogate 27,578 CpG sites and 482,421 CpG sites respectively [18]. Here we took a complementary approach to identify DNA methylation changes unique to the two endometrial cancer subtypes in an unbiased fashion. Our strategy allowed us to systematically measure DNA methylation levels of more than 20 million CpG sites in the cancer genome in an unbiased fashion with respect to intergenic and intronic regions, including repetitive regions derived from transposable elements. These regions are classically not measured by array-based methods, such as the ones employed by TCGA [19, 20].

We generated complete DNA methylome maps for endometrioid adenocarcinoma (EAC, three samples), uterine papillary serous carcinoma (UPSC, three samples), and normal endometrium (ten pooled samples) by integrating data from methylated DNA immunoprecipitation sequencing (MeDIP-seq) and methylation-sensitive restriction enzyme sequencing (MRE-seq) [19–24]. Comparative analysis of these seven DNA methylomes identified cancer-associated differentially methylated regions (DMRs) and distinct EAC and UPSC genomic DNA methylation patterns. Many methylation changes were found in CpG island shores and these changes were predicted to affect expression of nearby genes. We found demethylation across large domains on the X chromosome in UPSC accompanied by decreased *XIST* expression. We also identified methylation differences at numerous miRNA gene promoters that correlated with expression changes of the associated miRNAs. We discovered that cancer type-specific DMRs were enriched for not only promoters, but also for enhancer elements. Moreover, specific transposable elements (TEs), a rich genomic resource for potential enhancers [23, 25–32], were affected by both *de novo* methylation and demethylation in cancer samples. Together, these results suggest that DNA methylation broadly impacts cancer gene expression via regulation of promoters as well as enhancers and TEs.

## Results

### Distinct global and focal DNA methylation signatures in two types of endometrial cancer

DNA methylation changes have been reported in many types of cancers and exhibit strong tissue-specific and tumor type-specific characteristics [8, 33, 34]. DNA methylation changes in the two subtypes of endometrial cancers, endometrioid adenocarcinoma (EAC) and uterine papillary serous carcinoma (UPSC), were recently reported by The Cancer Genome Atlas Consortium [18]. This seminal study provided the first insight into DNA methylation changes at a genome-wide scale for this important cancer using array-based platforms. Here we took a different strategy, which is complementary to that of TCGA, to deeply profile complete DNA methylomes of a small number of tumor specimens using newly developed sequencing-based epigenomics technology [19, 20]. This strategy allowed us to systematically discover DNA methylation changes in cancer genomes without the biases imposed by microarray methods. Findings based on analyzing a small number of deeply profiled DNA methylomes (i.e., a discovery panel) can then be validated using array-based approaches, which can be applied to a much bigger number of samples (i.e., a validation panel). In this study, we generated complete DNA methylomes from six tumor samples (3 EACs and 3 UPSCs) and 1 pooled normal endometrium sample by applying methylated DNA immunoprecipitation sequencing (MeDIP-seq) and methylation-sensitive restriction enzyme sequencing (MRE-seq) [22]. We were able to comprehensively measure DNA methylation levels of more than 20 million CpGs for each sample (Additional file 1: Table S1). Sequencing data from the tumor samples were normalized by their genomic copy number variations identified using the Affymetrix SNP6.0 array.

Previous tumor DNA methylome studies have shown global demethylation accompanied by focal hypermethylation in many cancers [7–10]. To understand the global pattern of DNA methylation alterations in endometrial cancer, we directly compared the overall distribution of signal density from MeDIP-seq data at 5 kb resolution across the seven methylomes. As expected, cancer samples showed both hypomethylated and hypermethylated changes compared to normal endometrium (Additional file 2: Figure S1A). On average, 4.7% of the genome became hypomethylated, and 1.5% became hypermethylated in cancer (Additional file 1: Table S2). EAC and UPSC did not exhibit dramatic differences in this analysis.

**Figure 1:**
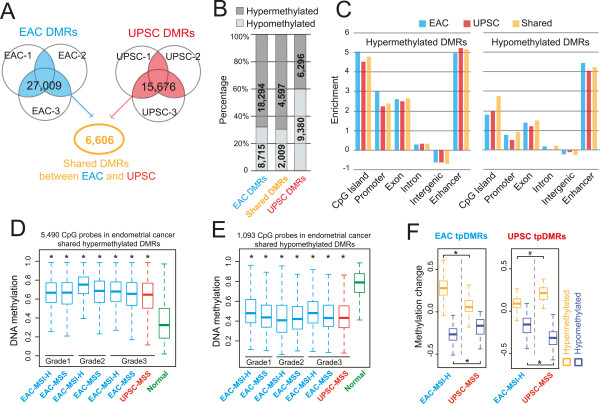
**Identification, annotation, and validation of DMRs in endometrioid adenocarcinoma (EAC) and uterine papillary serous carcinoma (UPSC). (A)** 27,009 DMRs were identified in at least 2 EAC samples (blue: EAC-DMRs), 15,676 DMRs were identified in at least 2 UPSC samples (red: UPSC-DMRs). 6,606 DMRs were identified in both EAC and UPSC as endometrial cancer (EC) shared DMRs (orange: EC-shared DMRs). **(B)** Percentage of hypermethylated (dark gray) and hypomethylated (light gray) EAC-, UPSC-, and EC-shared DMRs. **(C)** Genomic feature enrichment for hypermethylated DMRs (left) and hypomethylated DMRs (right). **(D)** The DNA methylation level of 5,490 CpGs located within 2,454 hypermethylated EC-shared DMRs in pre-classified (grade, microsatellite state, and subtype) endometrial cancer samples (blue: EAC; red: UPSC) and normal controls (green) in TCGA Infinium 450K data. DNA methylation level of each CpG site was averaged within the pre-classified group. Each boxplot represents the distribution of averaged methylation levels of 5,490 CpGs in cancer and controls. MSI-H: Microsatellite instability-high. MSS: Microsatellite stable. Asterisk: *P* < 1e-21 (Mann–Whitney *U* test). **(E)** The DNA methylation level of 1,093 CpGs located within 576 hypomethylated EC-shared DMRs in the same cohort (blue: EAC; red: UPSC) and controls (green) as in **(D)**. Asterisk: *P* < 1e-21 (Mann–Whitney *U* test). **(F)** Validation of EAC and UPSC type-preferred DMRs (tpDMRs) by TCGA Infinium 450K data. **Left**: DNA methylation change of 2,002 EAC tpDMRs in grade 3 MSI-H-EAC (blue) and MSS-UPSC (red), compared to normal controls. **Right**: DNA methylation change of 147 UPSC tpDMRs in same samples of UPSC (red) and EAC (blue). DNA methylation changes were calculated by subtracting the averaged methylation level of the normal controls from the methylation level of each DMR in each cancer sample. Asterisk: *P* < 1e-21, octothorpe: *P* <1e-3 (Mann–Whitney *U* test).

Our main goal was to identify local differentially methylated regions (DMRs) between cancer and normal endometrium in a genome-wide fashion. To this end, we developed and applied M&M, a novel algorithm that detects DMRs by integrating MeDIP-seq and MRE-seq data [19]. In total, we identified 27,009 EAC-associated DMRs and 15,676 UPSC-associated DMRs, with 6,606 DMRs in common between the two types of endometrial cancer (EC-shared DMRs) (Figure 1A and Additional file 1: Table S3). A complete list of these DMRs and links to the WashU Epigenome Browser were provided on the accompanying website (Methods). In EAC, 68% of DMRs (18,294) were hypermethylated and 32% (8,715) were hypomethylated relative to normal endometrium. In contrast, 40% (6,296) of the UPSC-associated DMRs were hypermethylated and 60% (9,380) were hypomethylated (Figure 1B). EAC and UPSC shared 4,597 hypermethylated and 2,009 hypomethylated DMRs (EC-shared DMRs). Previous studies have reported that two DNA methyltransferases (*DNMT1* and *DNMT3B*) were more highly expressed in EAC but repressed in UPSC [14, 17]. Upregulation of the DNMTs was confirmed by qRT-PCR in the three EAC samples we assayed. However, in the UPSC samples, *DNMT1*, *DNMT3A* and *DNMT3B* were not repressed, but had variable expression across samples (Additional file 2: Figure S1B). Consistent with our result, mRNA-seq data from TCGA also did not support previous studies: the three DNA methyltransfrases exhibited significantly higher expression in patients with UPSC as compared with non-tumor tissue (Additional file 2: Figure S1C).

The genomic distribution of cancer-associated DMRs was highly non-random (Additional file 1: Table S4). Over 70% of hypermethylated DMRs and 35% of hypomethylated DMRs were located in open chromatin regions and transcription factor binding sites, as defined by ENCODE [35] (Additional file 2: Figure S2A). Hypermethylated DMRs were strongly enriched for gene-related features, including CpG islands, promoters, and exons (Figure 1C). This is consistent with the notion that transcription-related regions, in particular CpG islands, are frequent targets of DNA methylation changes in cancer. However, these DMRs (CpG islands at promoters, exons, and UTRs) only accounted for 40% of the total hypermethylated DMRs in EACs. 60% of hypermethylated DMRs and 88% of hypomethylated DMRs were located in introns and intergenic regions (Additional file 2: Figure S2B). Strikingly, when we examined the relationship between these DMRs and annotated enhancers, we found that both hypermethylated and hypomethylated DMRs were strongly enriched for enhancer elements (Figure 1C). This suggests that in addition to promoters and CpG islands, enhancer elements can be a preferential target of DNA methylation alteration in cancers.

Next, we validated that the endometrial cancer-associated DMRs were recurrent by using Infinium 450K data produced by The Cancer Genome Atlas (TCGA). The Infinium 450K platform contains probes that span 6,583 CpG sites in 40% of 6,606 EC-shared DMRs (Additional file 2: Figure S2C). On average, over 85% EC-shared DMRs exhibited significant DNA methylation differences (Methods) between cancer patients and normal controls (Additional file 2: Figure S2D). Patients of all pathologic groups displayed consistent DNA methylation levels within these regions, robustly confirming our initial findings (Figure 1D and 1E). Importantly, these recurrent EC-shared DMRs exhibited hypo- or hypermethylation in low-grade EAC patients to the same degree as that of high-grade patients (Figure 1D and 1E), suggesting that abnormal DNA methylation is shared across tumors of different grades, and can potentially be detected in early stage of endometrial carcinogenesis. Thus, these EC-shared, recurrent DNA methylation changes could represent a unique epigenetic signature for endometrial cancers.

In addition, we defined cancer type-preferred DMRs (tpDMRs, see Methods). 3,443 EAC tpDMRs were present in all three EAC samples but not in any UPSC sample, and another 720 UPSC tpDMRs were present in all three UPSC samples but not in any EAC sample. Of these, 57% of EAC tpDMR regions and 21% of UPSC tpDMR regions contained Infinium 450K CpG probes (Additional file 2: Figure S2C), which allowed validation of our discovery using TCGA data. Among these regions, 86% of EAC tpDMRs and 89% UPSC tpDMRs showed significant DNA methylation differences (Methods) between cancer patents and normal controls (Additional file 2: Figure S2D). Additionally, EAC and UPSC tpDMRs were enriched in proximity to genes with different gene ontology terms (Additional file 2: Figure S3A). Among TCGA patient samples, tpDMRs were confirmed and found to exhibit cancer type-specific hypo- or hypermethylation status, suggesting that these cancer type-preferred DMRs are recurrent with respect to each cancer type (Figure 1F).

### Distinct DNA methylation patterns at CpG islands, shores, and gene promoters

There are about 28,000 CpG islands (CGIs) in the human genome. About 70% of gene promoters are associated with CGIs, and ~50% of CGIs contain annotated transcription start sites (TSS). The majority of promoter CGIs are unmethylated in somatic cells, although a significant portion of gene body CGIs exhibit a conserved, tissue-specific DNA methylation pattern [22, 35, 36]. DNA methylation of CpG islands is often correlated with the repression of the associated genes [37]. CpG islands frequently gain methylation in cancer [38]. Hypermethylation of CGIs, especially those associated with tumor suppressor genes, is considered a hallmark of many types of cancers [39, 40].

**Figure 2:**
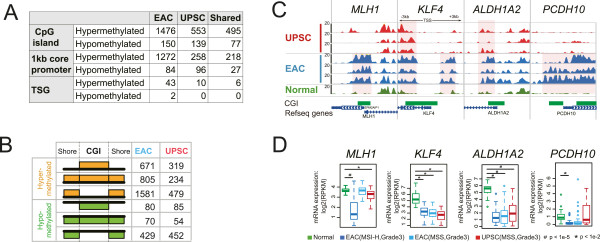
**Methylation changes at CpG islands and promoters. (A)** Numbers of CpG islands, 1 kb core promoter of RefSeq genes, and promoters of tumor suppressor genes (TSG) with hypermethylated and hypomethylated alterations in EAC and UPSC. **(B)** Classification of DNA methylation patterns in CpG islands and their shore regions: hypermethylation (orange) and hypomethylation (green) at CGI-only, both CGI and shores, and shores-only. **(C)** Epigenome Browser views of four tumor suppressor gene (TSG) promoters with different DNA methylation patterns across seven samples. MeDIP-seq tracks were displayed. The gene set view (-3 kb to +3 kb regions around TSS) was produced using the WashU Epigenome Browser. Increased methylation level was highlighted by pink shading. **(D)** Gene expression analysis of the same four TSG in **(C)** with hypermethylated promoters in normal controls and grade 3 pre-classified (microsatellite stability, subtype) endometrial cancers. Y-axis: RPKM value based on mRNA-seq from TCGA. Asterisk indicates *P* <0.01, octothorpe indicates *P* <1e-5, Student’s *t*-Test.

Examination of CGI DNA methylation changes in the two types of endometrial cancers revealed distinct signatures for EAC and UPSC. Compared to normal endometrium, we defined 1,476 and 553 hypermethylated CGIs for EAC and UPSC respectively (Figure 2A). 495 CGIs were hypermethylated in both types of endometrial cancer. 150 CGIs in EAC and 139 CGIs in UPSC exhibited reduced DNA methylation compared to normal endometrial cells. The two types of endometrial cancer can be clearly distinguished based on their CGI DNA methylation pattern (Additional file 2: Figure S2E).

Compared to promoter CGIs, non-promoter CGIs were more likely to undergo DNA methylation changes in endometrial cancers (Table 1). 532 CGIs (in EAC) and 193 CGIs (in UPSC) out of ~6,000 intergenic CGIs, and 448 CGIs (in EAC) and 223 CGIs (in UPSC) out of ~8,000 gene body CGIs, were hypermethylated. In contrast, of the ~13,000 promoter CGIs, only 496 and 137 were hypermethylated in EAC and UPSC respectively. Hypomethylated CGIs were also predominantly located in non-promoter regions, with 52 (in EAC) and 47 (in UPSC) in intergenic regions, and 94 (in EAC) and 85 (in UPSC) in gene bodies. Only 4 and 7 promoter CGIs were hypomethylated in EAC and UPSC respectively.

**Table 1:** **DNA methylation alterations of CpG islands in EAC and UPSC**

		Intergenic regions	Promoter	Gene-body
CpG islands (CGI) in human genome		6236	13212	8269
**EAC**	Hypermethylated CGI	532	496	448
	Hypomethylated CGI	52	4	94
**UPSC**	Hypermethylated CGI	193	137	223
	Hypermethylated CGI	47	7	85

Next, we examined DNA methylation alterations in CpG island shores (the flanking 1 kb around CGIs). Abnormal DNA methylation of CGI shores was first reported in colon cancer and found to affect expression of the nearby genes [8]. In endometrial cancer, CGI shores exhibited dynamic DNA methylation changes that were independent of changes in the CpG islands (Figure 2B). We further classified DNA methylation changes with respect to their patterns in both CGIs and corresponding shores. In EAC, 805 of 1,476 hypermethylated CGIs also displayed hypermethylation in their shore regions, while 234 of 553 UPSC hypermethylated CGIs had accompanying hypermethylated shores. Strikingly, 1,581 EAC CGIs and 479 UPSC CGIs without significant DNA methylation changes had flanking hypermethylated shores. Additionally, 429 CGIs in EAC and 452 CGIs in UPSC were specifically hypomethylated in their shore regions but not in CGIs themselves (Figure 2B). In EAC, 791 genes, including lncRNA genes, were found around these 1,581 CGI shore-only hypermethylated CGIs, and 116 genes were close to 429 shore-only hypomethylated CGIs.

We then examined the DNA methylation status of 1 kb core promoters around the TSS of annotated RefSeq genes (Figure 2A). 1272 and 84 genes were hypermethylated or hypomethylated, respectively, at their core promoters in EAC. These numbers were 258 and 96 in UPSC. This result highlights the difference between the two types of endometrial cancers. 218 gene promoters were hypermethylated in both tumor types, while 27 gene promoters were hypomethylated in both. Gene function enrichment analysis of these promoters returned no significantly enriched terms related to endometrial tissue function (Additional file 2: Figure S3B).

We observed 43 tumor suppressor genes (TSGs) [41] with hypermethylated promoters in EAC, which is consistent with our expectation that the silencing of tumor suppressor genes is an important component of carcinogenesis. In contrast, in UPSC we found only 10 tumor suppressor genes with hypermethylated promoters (Additional file 2: Figure S4 and S5). As expected, the majority of these tumor suppressor genes were repressed in EAC (Additional file 2: Figure S4 and S5). Surprisingly, some of these tumor suppressor genes were also repressed in UPSC, even though the promoter regions were unmethylated as in normal endometrium. For example, a well-known marker, the promoter of the *MLH1* gene, is usually highly methylated in microsatellite instability-high (MSI-H) EAC, and was unmethylated in UPSC (Figure 2C and Additional file 2: Figure S5C). However, RNA-seq data from TCGA suggested that expression of *MLH1* was also repressed in some UPSC patients (Figure 2D). As expected, hypermethylation of tumor suppressor gene promoters was strongly associated with gene repression. For example, *KLF4* had a hypermethylated CGI shore and gene body and was repressed in three groups of endometrial cancer patients (Figure 2D). Similarly, the promoter of *ALDH1A2* was hypermethylated and expression of this gene was repressed in both EAC and UPSC. In contrast, the promoter of *PCDH10* was specifically methylated in EAC, and this gene was respressed in EAC only (Figure 2C and 2D). Unexpectedly, we found the core promoter regions of two tumor suppressor genes, *CDH1* and *SFN*, were demethylated in three EAC samples (Additional file 2: Figure S6A). Interestingly, these two genes were significantly upregulated in all three groups of endometrial cancer patients from TCGA (Additional file 2: Figure S6B). *CDH1* and *SFN* are important tumor suppressor genes whose loss of function has been implicated in many types of cancers [42–45]. Thus, their demethylation and increased expression underscores the complexity of regulatory events in endometrial cancer.

### Loss of X chromosome DNA methylation and *XIST*expression in UPSC

**Figure 3:**
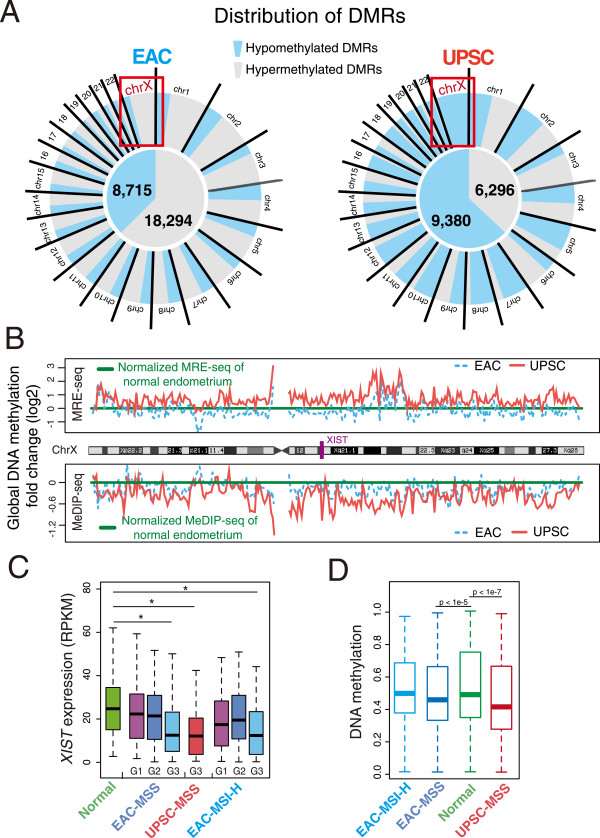
**Loss of DNA methylation on the X chromosome. (A)** Distribution of hypermethylated (gray) and hypomethylated DMRs (blue) on all chromosomes in EAC (left) and UPSC (right). **(B)** Global DNA methylation changes on the X chromosome. MeDIP-seq and MRE-seq RPKM values of seven samples were calculated at 500 kb resolution across the X chromosome. The averaged RPKM fold changes (cancer/normal) of each type (3 EACs and 3 UPSCs) were log2-transformed and plotted along with the X chromosome coordinate. **(C)** *XIST* expression in normal controls and pre-classified (grade: G1, G2, G3; microsatellite state, and subtype) endometrial cancer samples. Y-axis: calculated RPKM value based on mRNA-seq from TCGA. Asterisk indicates *P* <0.01, Student’s *t*-test. **(D)** DNA methylation distribution of 9,620 CpG sites on the X chromosome in pre-classified grade 3 (microsatellite state, and subtype) uterine corpus endometrial cancer samples (light blue: MSI-H type EAC patients; dark blue: MSS type EAC patients; red: MSS type UPSC patients. Infinium 450K platform) and normal controls (green, Infinium 450K platform). For each CpG site, the averaged DNA methylation level was calculated within the pre-classified group, and each boxplot represents distribution of the averaged methylation level of all 9,620 CpGs of the cancer groups and the normal controls. CpG sites with no value in any sample were removed. MSI-H: Microsatellite instability-high. MSS: Microsatellite stability. The Mann–Whitney *U* test was performed respectively for each cancer group when compared to normal controls.

One prominent signature distinguishing EAC and UPSC was DNA methylation differences on the X chromosome. More than 95% of DMRs on chromosome X were hypomethylated in UPSC (Figure 3A and Additional file 1: Table S5). In contrast, only ~16% of chromosome X DMRs were hypomethylated in EAC. Plotting MeDIP-seq signal and MRE-seq signal at a large scale (500 kb resolution) revealed loss of DNA methylation across the whole X chromosome in UPSC, as evidenced by reduced MeDIP-seq signal (~20%) and increased MRE-seq signal (~60%) (Figure 3B). EAC also exhibited loss of DNA methylation across the X chromosome, but to a much lesser degree than UPSC. In contrast, similar analysis of any autosome (e.g., chromosome 10) revealed no global change of DNA methylation in either EAC or UPSC (Additional file 2: Figure S7). We further examined expression of *XIST*, the lncRNA critical for establishing inactivation of the X chromosome, both in published data and by performing qRT-PCR on our samples. *XIST* showed reduced expression in both EAC and UPSC. Interestingly, in microsatellite stable (MSS) type EAC samples, the expression level of *XIST* was inversely correlated with the tumor grade (cor = -0.19) (Figure 3C). qRT-PCR also confirmed that *XIST* was down-regulated in four of six endometrial cancer samples in our study (Additional file 2: Figure S1D). Finally, we examined the methylation level of 9,620 CpGs (~0.8% of total CpGs on the X chromosome) in TCGA endometrial cancer samples profiled on the Infinium 450K platform (Figure 3D). As expected, compared to normal controls (mean 0.52), the patients with grade 3 UPSC cancer type showed significantly lower DNA methylation level (mean 0.45, p-value < 1e-7), and the patients with grade 3 MSI-H EAC cancer type did not show difference on these CpG sites (mean 0.51, p-value = 0.21). Interestingly, the TCGA samples with grade 3 MSS EAC cancer type also show lightly reduced DNA methylation level on the X chromosome (mean 0.48, p-value < 1e-5). These results suggest that global hypomethylation of X chromosome and repressed *XIST* expression constitute a strong signature for UPSC cancers.

### DNA methylation changes of non-coding RNA genes

Our genome-wide DNA methylome maps also allowed us to comprehensively assess DNA methylation changes around non-coding RNA genes, including miRNA and lncRNA. Epigenetic changes in promoters of non-coding RNA genes could result in changes of their expression levels, which subsequently could result in expression changes of downstream protein coding genes in endometrial cancer [46] and in changes of the epigenetic landscape [47].

We examined DNA methylation patterns in promoter regions around the TSS of miRNA gene clusters [48]. In EAC, promoters of 24 miRNA gene clusters exhibited increased DNA methylation, while 6 miRNA gene clusters exhibited reduced DNA methylation. These two numbers in UPSC were 9 and 2 (Additional file 2: Figure S8A). Some of these miRNAs were reported to have significant expression changes in endometrial cancer [46, 49–51]. By examining TCGA miRNA-seq data, we found that expression levels of certain miRNAs correlated with DNA methylation changes in their promoters (Additional file 2: Figure S8B and S8C). The microRNA cluster *MIR200B-MIR200A-MIR429* and cluster *MIR200C-MIR141* showed significant demethylation in endometrial cancer, and the expression levels of these five microRNAs were significantly increased in three groups of endometrial cancer patients (Additional file 2: Figure S8C and S8D). The *MIR200* family was considered a tumor suppressor because of their inhibitory role in epithelial-mesenchymal transition (EMT) in bladder cancer and breast cancer cell lines [52–54]. Moreover, high expression of this cluster was tightly correlated with survival rate in those tumor types, and overexpression of these miRNAs inhibited ovarian cancer cell motility [55]. In addition, the *MIR200* family was reported to show significant upregulation in endometrial carcinomas [50, 56, 57]. The exact functions of *MIR200* family members in gynecologic cancers, especially in endometrial carcinogenesis, still need to be determined. Interestingly, even with strongly hypermethylated promoters, some miRNAs exhibited upregulated expression level, including *MIR25*, *MIR93*, *MIR99B*, *MIR324*, *MIR106B*, and *MIR3074* (Additional file 2: Figure S8E). The regulatory relationship between promoter hypermethylation and microRNA expression is likely complex and needs further investigation.

Hundreds of lncRNA genes [58] also underwent DNA methylation changes in our endometrial samples (Additional file 2: Figure S8A). Specifically, 621 and 168 lncRNA genes had hypermethylated promoters in EAC and UPSC respectively, compared to normal endometrium. Of these, 144 were in common between the two cancer types. The numbers of hypomethylated lncRNA genes were 207 for EAC and 245 for UPSC, with 68 in common. With the exception of classic examples such as *XIST*, *H19*, and *HOTAIR*, the widespread regulatory roles of lncRNAs are only recently being revealed [59]. The roles of lncRNAs and consequences of their abnormal DNA methylation in endometrial carcinogenesis remain to be elucidated. However, interesting candidate genes emerged from our analysis. For example, maternally expressed gene 3 (*MEG3*), a tumor suppressor non-coding RNA [60], was highly methylated at its promoter region in EAC. This was associated with significantly downregulated *MEG3* mRNA (Additional file 2: Figure S9). Re-expression of *MEG3* was found to induce *TP53* activation, and inhibited tumor cell proliferation in culture and colony formation in soft agar [60–64].

### DNA methylation changes were enriched in functional promoter and enhancer elements

**Figure 4:**
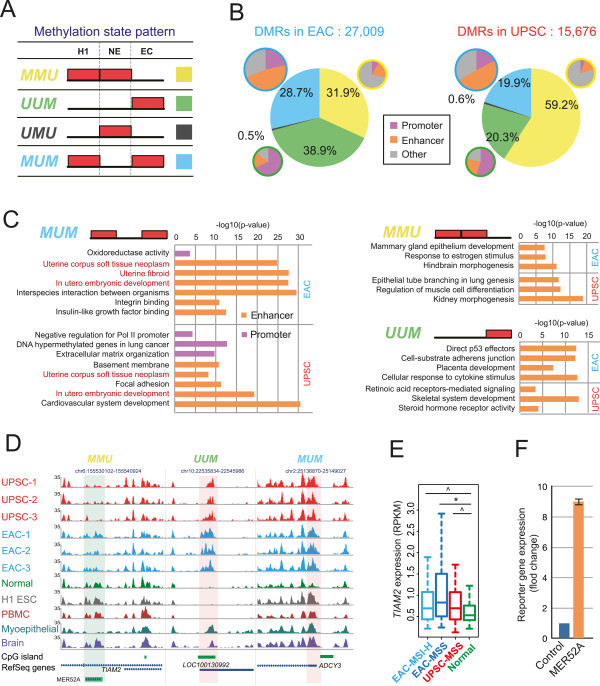
**DNA methylation alterations are enriched in functional promoters and enhancers. (A)** Four DNA methylation state patterns across embryonic stem cells (H1), normal endometrium (NE), and endometrial cancer (EC). Red block: methylated state; empty: unmethylated state. **(B)** Fractions of DMRs with different DNA methylation state patterns in EAC (left) and UPSC (right). DMRs with patterns ***MMU*** (yellow), ***UUM*** (green), and ***MUM*** (blue) were annotated by chromHMM for their potential regulatory functions (purple: promoter; orange: enhancer state; gray: other states). **(C)** Functional enrichment of DMRs in pattern ***MMU***, ***UUM***, and ***MUM*** by GREAT analysis (purple: DMRs annotated as promoters by chromHMM; orange: DMRs annotated as enhancers by chromHMM). X-axis denotes negative log10-transformed p-value. **(D)** Epigenome Browser views of representative DMRs with different methylation state patterns. MeDIP-seq signal tracks were displayed. Increased and decreased methylation levels were highlighted by pink and green shading, respectively. **Left** (pattern ***MMU***): a DMR overlapped with transposable element *MER52A* was methylated in normal endometrium, H1 ESC, PBMC, and fetal brain, but unmethylated in breast myoepithelial cells. In EAC and UPSC, the element was hypomethylated, indicated by decreased MeDIP-seq signal. **Middle** (pattern ***UUM***): a DMR overlapped with CGI of lncRNA *LOC100130992* was unmethylated in normal endometrium, H1 ESC, and PBMC, but methylated in breast myoepithelial and fetal brain. The element was hypermethylated in EAC and UPSC. **Right** (pattern ***MUM***): a DMR overlapped with CGI-shore of *ADCY3* was methylated in all other tissues except in normal endometrium. In EAC and UPSC, this element was hypermethylated. **(E)** *TIAM2* expression in normal controls and grade 3 pre-classified (microsatellite state, and subtype) endometrial cancer patients samples. Y-axis: calculated RPKM value based on mRNA-seq from TCGA. Asterisk indicates *P* <0.01, caret indicates *P* <0.05, Student’s *t*-Test. **(F)** The *MER52A* element enhanced luciferase reporter gene expression in HEK-293T cells. Blue: mini promoter; orange: *MER52A* + mini promoter.

The majority of the differentially methylated regions were located in intergenic or intragenic regions, while a small fraction (11% in EACs, and 5% in UPSCs) overlapped directly with annotated gene promoters. Previous work reported that normal tissue developmental processes could be used to model tumor developmental states [65]. Here we examined endometrial cancer DMRs in the context of their epigenetic status in embryonic stem cell (ESC) H1 to map the dynamics of DNA methylation changes [19]. We defined four DNA methylation patterns across ESC, normal endometrium, and endometrial cancer (Figure 4A): ***MMU***, **M**ethylated in ES cells, **M**ethylated in normal endometrium, but **U**nmethylated (or hypomethylated) in cancer; ***MUM***, **M**ethylated in ES cells, **U**nmethylated in normal endometrium, and **M**ethylated in cancer; ***UMU***, **U**nmethylated in ES cells, **M**ethylated in normal endometrium, and **U**nmethylated in cancer; and ***UUM***, **U**nmethylated in ES cells, **U**nmethylated in normal endometrium, and **M**ethylated in cancer. We reasoned that the genomic distribution and functions of genes associated with different patterns might help to reveal insights into endometrial carcinogenesis in the context of normal development. We annotated the potential regulatory function of these different categories of DMRs with the chromatin state maps defined by chromHMM using nine cell lines [66]. Interestingly, the majority of the ***UUM*** DMRs were annotated as promoters, whereas the majority of the ***MUM*** DMRs were annotated as enhancers (Figure 4B).

We then examined the functional enrichment of genes near these four different categories of DMRs (Figure 4C and Additional file 2: Figure S10A). Significantly, genes near pattern ***MUM*** enhancer DMRs in both types of cancer were strongly enriched for terms related to uterine development and disease. Consistent with our previous annotation of tissue-specific enhancers [19], this result suggested that these DMRs might encode uterine-specific regulatory elements that became unmethylated and activated during normal development and differentiation. Their hypermethylation in endometrial cancer might contribute to the loss of endometrial tissue type identity and to the gain of stem cell-like characteristics often observed in cancers. In contrast, pattern ***MMU*** DMRs enriched for functions specific to tissues unrelated to endometrium. For example, in EAC, genes related to hindbrain morphogenesis and mammary gland epithelium development were enriched near ***MMU*** DMRs. In UPSC, we found enrichment of genes related to lung, muscle, and kidney development. In addition, we found genes related to estrogen response, consistent with the strong connection between EAC and estrogen [67, 68]. This data suggested a very different but complementary mechanism for cancer gene deregulation: these ***MMU*** DMRs might represent a broad array of tissue- or cell type-specific regulatory sites important for development and differentiation. Their demethylation in endometrial cancer might simultaneously deregulate tissue-specific genes important for other tissues, thereby contributing to the loss of endometrium specificity. The ***UUM*** pattern, on the other hand, was associated with functions related to carcinogenesis in general, including RNA polymerase II, nucleic acid-binding transcription factors, tumor suppressor genes, direct effectors of *TP53*, cell-substrate adherent factors, and agents of cellular response to cytokine stimuli (Figure 4C and Additional file 2: Figure S10A). Finally, very few DMRs exhibited the ***UMU*** pattern, which was predicted to be associated with embryonic development (Additional file 2: Figure S10A).

We highlighted a few examples with different methylation patterns (Figure 4D). A DMR with pattern ***MMU*** overlapped a copy of transposable element *MER52A*. This DMR was predicted to be an enhancer by chromHMM [66] and was located in the intron of *TIAM2* and 2.7 kb upstream of a gene-body alternative promoter. The DMR was highly methylated in ESC and normal endometrium, but was hypomethylated in endometrial cancer, particularly in UPSC. This element was also hypomethylated in breast myoepithelial cells but methylated in blood cells and brain tissues, consistent with the hypothesis that ***MMU*** pattern was associated with tissue-specific enhancers (Figure 4D). *TIAM2* regulates focal adhesion, and knockdown of this gene leads to a reduced rate of cell migration [69]. By examining TCGA mRNA-seq data, we found that the expression level of *TIAM2* was upregulated in both EAC and UPSC, compared to normal controls (Figure 4E). Importantly, we did not find DNA methylation changes at the promoter region of *TIAM2*. Based on a luciferase reporter assay, this *MER52A* element increased reporter gene expression about 9-fold in HEK-293T cells (Figure 4F). These results suggested that demethylation of this transposable element could activate its enhancer function and increase *TIAM2* expression, potentially contributing to cancer cell migration and endometrial carcinogenesis. In another example, a pattern ***UUM*** DMR was identified in the promoter region of lncRNA *LOC100130992*. The DNA methylation status of this promoter was tissue-specific: methylated in breast myoepithelial cells and brain tissue, but unmethylated in H1 ESC, normal endometrium cells, and peripheral blood monocytes (Figure 4D). Numerous promoter DMRs with pattern ***MUM*** were predicted to play roles in normal endometrial functions. For example, the *ADCY3* promoter was specifically hypomethylated in normal endometrium, but its associated CpG island shore was highly methylated in EAC and UPSC. *ADCY3* encodes adenylyl cyclase 3, which catalyzes the formation of the secondary messenger cyclic adenosine monophosphate (cAMP). cAMP stimulates vascular endothelial growth factor (*VEGF*) secretion by human endometrial stromal cells [70] and mediates progesterone-dependent decidualization of the human endometrium [71]. *ADCY3* expression was indeed repressed in both EAC and UPSC (Additional file 2: Figure S10B).

### Bidirectional DNA methylation alteration on TE-derived enhancers

**Figure 5:**
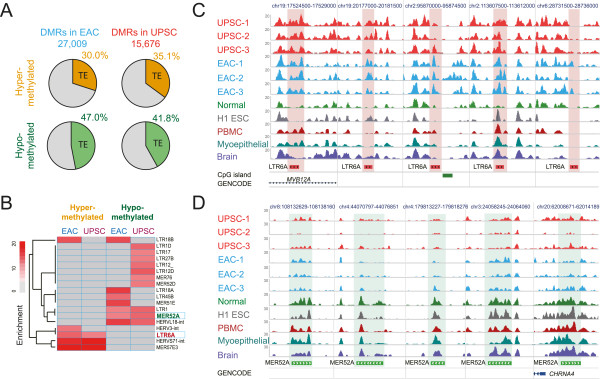
**DNA methylation alterations of transposable elements. (A)** Percentage of DMRs overlapping transposable elements in EAC and UPSC. **(B)** DMR enrichment within transposable element subfamilies. Enrichments > 5-fold were shaded in red. **(C)** Epigenome browser view of five genomic copies of *LTR6A* and their methylation levels across multiple samples. Only MeDIP-seq signal tracks were included. Increased methylation levels were highlighted by pink shading. These elements were unmethylated in normal endometrium, differentially methylated across H1 ESC, PBMC, breast myoepithelial cells, and fetal brain, and hypermethylated in EAC and UPSC. **(D)** Epigenome browser view of five genomic copies of *MER52A* and their methylation levels across multiple samples. MeDIP-seq signal tracks were displayed. Decreased methylation levels were highlighted by green shading. These elements were methylated (normal endometrium, H1 ESC, PBMC, and fetal brain) or partially methylated (breast myoepithelial cells) in normal cells, but were hypomethylated in EAC and UPSC.

We noted that the *TIAM2* intragenic enhancer was within a transposable element. TEs make up nearly half of the human genome, but they have long been considered “junk DNA” that does not systematically contribute to normal cellular function [25, 72]. Deregulation of TEs has, however, been proposed to be an important contributor to carcinogenesis. TEs, especially LINE-1 retrotransposons, are hypomethylated in many types of cancers [73–78]. We systematically evaluated the DNA methylation state of all TEs in the human genome that could be uniquely mapped by our sequencing technology. Surprisingly, more than 30% of hypermethylated DMRs and more than 40% of hypomethylated DMRs in both types of endometrial cancer were within TEs (Figure 5A). These changes in methylation status were enriched for specific TE families and subfamilies. For example, the LINE-1 family was hypomethylated in both EAC and UPSC (Additional file 2: Figure S11), consistent with observations made in other cancer types [73–75]. We calculated enrichment of TE subfamilies in endometrial cancer DMRs. Four subfamilies were significantly more likely to be hypermethylated in EAC or UPSC (enrichment > 5-fold), whereas 13 subfamilies were significantly more likely to be hypomethylated in EAC or UPSC (enrichment > 5-fold) (Figure 5B).

We further investigated individual TE copies that exhibited a change in DNA methylation. For the purpose of illustration, we used *LTR6A* and *MER52A*. We computed methylation levels of individual copies of *LTR6A* and *MER52A* in EAC and UPSC (RPKM values from MeDIP-seq and MRE-seq data normalized to the values in normal endometrium). About 70% of *LTR6A* elements exhibited increased MeDIP-seq and decreased MRE-seq signal, reflecting hypermethylation (Additional file 2: Figure S12A). In contrast, around 70% of *MER52A* elements had decreased MeDIP-seq and increased MRE-seq (Additional file 2: Figure S12B). The most highly hypermethylated *LTR6A* copies were found to be differentially methylated across different tissues, and were annotated as enhancers by chromHMM (Figure 5C). In contrast, the most highly hypomethylated *MER52A* copies exhibited strong methylation in all five normal tissues (including H1 ESC, breast, blood, brain, and endometrium) (Figure 5D).

Our results indicate that TEs may be important regulatory elements, particularly enhancers, in carcinogenesis. Transposable elements have been shown to wire gene regulatory networks during evolution [25, 27–30], and TE subfamilies with tissue-specific hypomethylation also function as enhancers, which may associate with tissue identity [23]. How these transposable elements contribute to endometrial carcinogenesis remains to be elucidated. Moreover, in contrast to the common belief that most TEs are methylated and repressed in somatic cells [75, 79, 80], the discovery that a significant fraction of TEs were unmethylated in normal tissues but methylated in cancer suggests that some TEs play important roles in maintaining normal cell states and sustaining regular cellular physiological processes. These TE-mediated processes were likely disrupted in cancer cells. The widespread abnormal DNA methylation of transposable elements in cancer could reflect rewiring of gene regulatory networks during carcinogenesis. In normal tissues, some TEs were unmethylated and acted as tissue-specific enhancers. When these TEs were hypermethylated in cancer cells, they were silenced and this could have contributed to the down-regulation or silencing of target genes. On the other hand, hypomethylation of normally methylated TEs could function as cryptic enhancers or promoters [20], contributing to the upregulation of target oncogenes.

## Discussion

**Figure 6:**
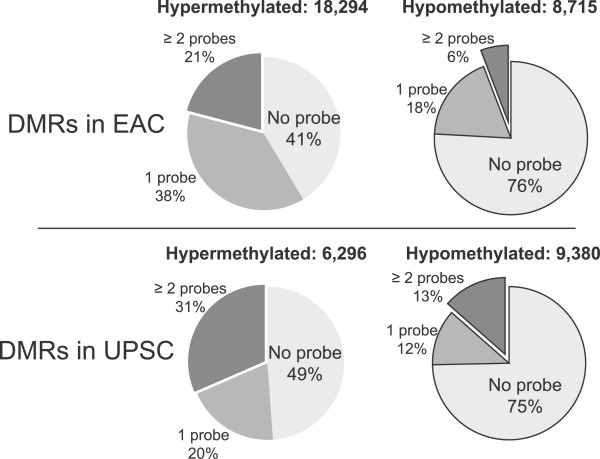
**Infinium 450K array probe representation for DMRs.** For EAC DMRs, 41% of hypermethylated and 76% of hypomethylated regions were not represented by Infinium 450K probes (light gray). For the UPSC DMRs, 49% of hypermethylated and 75% of hypomethylated regions did not contain Infinium 450K probes (light gray).

Changes in DNA methylation have been shown to play roles in carcinogenesis and cancer progression in many malignancies [4, 8, 9] including endometrial cancer [11, 12]. Previous studies have identified many genes exhibiting abnormal DNA methylation changes in endometrial cancer [11–14, 16, 81]; however, these studies, including the most recent and comprehensive mapping of DNA methylation in endometrial cancers by TCGA [18], were all focused on selected genomic regions (e.g., promoters and CpG islands) and CpG sites. Our study systematically investigated the complete DNA methylome in two clinically distinct types of endometrial cancer, EAC and UPSC, as well as normal endometrium, providing the first whole-genome DNA methylation map for this important disease. We observed significant DNA methylation changes in both types of endometrial cancer, which displayed both shared and cancer subtype-preferred DNA methylation signatures. Endometrial cancer-shared DMRs defined a specific signature of endometrial cancer that is confirmed by TCGA endometrial cancer methylation data (Figure 1D and 1E). In UPSC, which previously was thought to have infrequent DNA methylation changes and low *DNMT* expression, we identified numerous novel DNA methylation alterations (95% of 15,657 UPSC DMRs) in non-promoter regions (intergenic and intragenic). These results demonstrate the importance of applying sequencing-based, whole-genome approaches (i.e., MeDIP-seq and MRE-seq) to comprehensively map DNA methylation changes in cancers. In contrast, the Infinium HumanMethylation450 BeadChip, a popular genome-wide platform used by TCGA and many others, only interrogates about 1.7% (485,000) of the 28 million CpGs in the human genome. In our study, more than 40% of the hypermethylated DMRs and 75% of the hypomethylated DMRs we identified were not covered by any Infinium probes; additionally only about 25% of the hypermethylated DMRs and 9% of the hypomethylated DMRs had more than two probes (Figure 6).

Despite being much more comprehensive, sequencing-based DNA methylome technologies are still cost inhibitory for directly profiling the large cohort of specimens that is often required to obtain power to detect most epigenetic events significantly associated with cancer. Thus, we advocate a strategy that combines deep profiling of a small number of samples (in this study, three samples per cancer type) followed by validation in a large cohort (in this study, we used publically available TCGA data). The strong validation results (Figure 1D-1F) suggest that our strategy is able to capture recurrent abnormal DNA methylation and provide a much more complete picture of the epigenetic landscape in endometrial cancer.

CpG islands and gene promoters are classic targets of DNA methylation changes in carcinogenesis. In endometrial cancer, we confirmed that hypermethylation was strongly enriched in transcription-related regions, including CGIs and promoters. Over 65% of CGIs with altered methylation were located in intergenic and intragenic regions, suggesting that alternative promoters may be frequent targets of DNA methylation change in cancers [20, 22]. In addition, CGI shores were frequently affected by DNA methylation alterations (Figure 2A and Additional file 2: Figure S6A) [8, 34]. Similar to CGIs, epigenetic changes at these potential regulatory regions may also regulate expression of nearby genes (Figure 2D, Additional file 2: Figure S6B). Epigenetic silencing of tumor suppressor genes (TSGs) is an important mechanism of carcinogenesis. In our study, many TSGs had hypermethylation in their core promoter regions, but interestingly, two tumor suppressor genes (*CDH1* and *SNF*) were found to have hypomethylated promoters and increased mRNA expression. Hypomethylation of the *S*NF promoter was also reported in lung and prostate cancer [42, 43]. However, the methylation pattern of *CDH1* seems to be much more complex in endometrial cancer. Hypermethylated *CDH1* promoter and repressed *CDH1* expression were reported in some endometrial cancer cases [82, 83]. Our approach revealed a hypomethylated DMR located in the 5’ shore region of the *CDH1* promoter CpG island. This discovery was confirmed by TCGA Infinium 450K data: significant demethylation was detected by 2 CpG probes located in the DMR, but 8 nearby CpG probes within the CpG island did not detect any DNA methylation change (Additional file 2: Figure S6C). These results suggest that a demethylated promoter and increased expression of *CDH1* might be a common feature of endometrioid adenocarcinomas. The role of *CHD1* in endometrial cancer remains to be determined.

During human embryonic development, one copy of the X chromosome is randomly inactivated in female cells. The X-inactivation process is initiated by the expression of *XIST*, a mono-allelically expressed long non-coding RNA. High allelic expression of *XIST* on the inactive X establishes the inactivation state [84–88]. DNA methylation and repressive histone modifications, such as H3K27me3 and H3K9me3, play essential roles in maintaining the inactivated state [85, 89–93]. The link between X chromosome epigenetic changes and female reproductive cancers was evident in a subset of breast and ovarian cancers [94]. In UPSC, we observed significant demethylation across the entire X chromosome, accompanied by down-regulated *XIST* expression. Interestingly, the expression level of *XIST* was also decreased in EAC and correlated with tumor grade (Figure 3C). In contrast to UPSC, we did not observe a significant global hypomethylation pattern in our three EAC samples. TCGA data revealed no global changes in the MSI-H type EAC, but a small but significant global hypomethylaton in the MSS type of EAC and in UPSC (Figure 3D). The Infinium 450K array only interrogates ~0.8% CpG sites on the X chromosome and is biased towards CpG sites in promoters and CpG islands. Thus, further validation is necessary to confirm the status of X chromosome DNA methylation. However, our results suggest that this significant sex chromosome demethylation could be a distinguishing signature between EAC and UPSC and might be associated with microsatellite stability. In ovarian cancer, a strong association was found between down regulated *XIST* expression and decreased time to recurrence in patients treated with Taxol [95]. Although the mechanisms underlying *XIST* down-regulation and the biological significance of DNA methylation loss remain to be determined, we hypothesize that repressed *XIST* expression and DNA demethylation might contribute to the aggressive behavior in endometrial cancer, especially in UPSC.

Up to 80% of the human genome is transcribed but only ~2% encodes proteins [96–98]. In the past decade, numerous small and long non-coding RNAs (lncRNA) have been shown to be important regulators in both normal development and disease [59, 99]. We observed DNA methylation alterations at many miRNA promoters. The expression of some miRNAs was correlated with DNA methylation states. For example, the *MIR200A-MIR200B-MIR429* cluster, which are tumor suppressor genes in many cancers, showed strong promoter demethylation in EAC and UPSC, with significantly upregulated expression (Additional file 2: Figure S8C and S8D). We also observed DNA methylation alterations at promoters of hundreds of lncRNA genes. The precise biological function of these miRNAs and lncRNAs and the significance of their DNA methylation changes in endometrial cancers remain to be determined. However, their identification underscores the importance of having a comprehensive catalog of cancer-associated DNA methylation changes across the whole genome.

DNA methylation exhibits strong tissue specificity, which is tightly programmed during embryonic development [100–104]. Alteration of DNA methylation in cancer radically changes the normal epigenetic landscape and results in loss of tissue specificity or cell identity [40]. For example, colorectal cancer DMRs had a striking overlap with tissue-specific DMRs located in CGI shore regions, possibly disrupting tissue-specific regulatory programs [8]. By including DNA methylome data from embryonic stem cells, we identified a subset of endometrial cancer DMRs showing specific demethylation in normal endometrium, but high methylation in both H1 ESC and endometrial cancer (DNA methylation pattern ***MUM***). These DMRs, which accounted for 30% of EAC DMRs and 20% of UPSC DMRs, showed a strong functional enrichment for genes related to uterine embryonic development and disease. Moreover, more than half of these ***MUM*** DMRs and ~25% of ***MMU*** and ***UUM*** DMRs were predicted to be distal enhancers (Figure 4B) that were deregulated in endometrial cancers. These results highlight that in addition to gene promoters, enhancers are a major target of DNA methylation changes in endometrial cancer.

**Figure 7:**
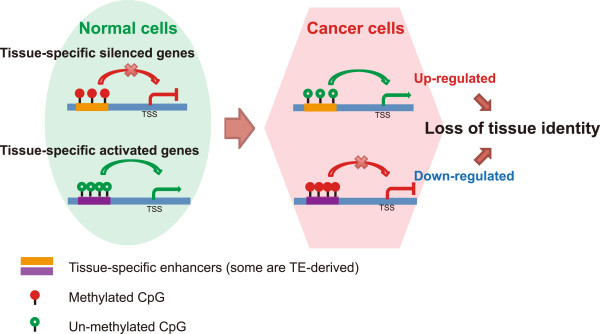
**Proposed model for loss of tissue identity during carcinogenesis associated with epigenetic alterations at enhancers. Left**: Normal DNA methylation at enhancers mediates tissue-specific gene silencing and activation in normal cells. **Right**: DNA methylation alteration at enhancers in cancer cells induces abnormal regulation and subsequent changes in expression of tissue-specifically silenced or activated genes, leading to loss of tissue identity.

Interestingly, a significant fraction of deregulated enhancers were derived from transposable elements (TEs): more than 30% of hypermethylated and 40% of hypomethylated DMRs harbored TEs. TEs have classically been understood to have high methylation in normal somatic cells and undergo global demethylation in cancers [10, 73–75]. However, recent data have demonstrated that TEs play many important roles in normal biology, especially in wiring gene regulatory networks during evolution [25, 27–30], and contributing to the establishment of tissue-specific gene regulation in development [23]. A breast-specific hypomethylated LTR element, *MER52A*, was found in the intron of the oncogenic *TIAM2* gene. This element was methylated in normal endometrium but demethylated in endometrial cancer (Figure 4D), and its enhancer function was validated using a reporter gene assay (Figure 4F). Our study suggested that DNA methylation in TEs might have a much more profound impact in cancer than previously believed. Consistent with our recent discovery [23], we identified unmethylated TEs in normal endometrium that may serve as tissue-specific enhancers. Some of these TE-derived, tissue-specific enhancers were hypermethylated and presumably silenced in endometrial cancer samples. In contrast, some normally methylated TEs (and TE-derived enhancers, possibly specific for other tissues) became hypomethylated in endometrial cancer. Disruption of normal DNA methylation of TE-derived and tissue-specific enhancers might be a novel mechanism to facilitate loss of tissue identity, acquisition of new cell type phenotypes, and to contribute to tumor development and progression (Figure 7). Complex rewiring of gene networks by epigenetic alterations might play a critical role in endometrial carcinogenesis. However, additional data, including a comprehensive annotation of the enhancer landscape in normal endometrial cells and in endometrial cancers, are needed to further elucidate this important mechanism.

## Conclusions

We systematically investigated the complete DNA methylome in two clinically distinct types of endometrial cancer, EAC and UPSC, as well as normal endometrium, providing the first whole-genome DNA methylation map for this common and deadly disease. From these datasets, we identified tens of thousands of DMRs specific to the two cancer subtypes (endometrioid adenocarcinoma, or EAC, and uterine papillary serous carcinoma, or UPSC), and common to both cancer subtypes. We estimate that more than 2/3 of the DMRs we identified could not be discovered by using current array-based platforms. Many methylation changes were found in CpG island shores and were associated with expression changes of nearby genes. We observed large-scale DNA demethylation of chromosome X in UPSC accompanied by decreased *XIST* expression. Most significantly, we discovered that the majority of DMRs harbor regulatory functions including promoters and enhancers that are important to developmental and pathological changes of the uterus. Among these, remethylation of transposable elements in cancers might provide a novel mechanism to deregulate normal endometrium-specific enhancers derived from specific transposable elements. Our results demonstrate that DNA methylation changes are an important signature of endometrial cancer and regulate gene expression by affecting not only proximal promoters, but also distal enhancers, including those derived from transposable elements.

## Methods

### Website

A complete list of endometrial cancer associated DMRs and links to the WashU Epigenome Browser [105, 106] are provided at the following website:

http://epigenome.wustl.edu/Cancer_Epigenome/.

### Sample collection

All primary endometrial tumors and normal endometrium specimens analyzed were collected as part of IRB-approved studies (Washington University Medical Center Human Research Protection Office protocols HRPO-91-507, -93-0828, -92-242, and -10-1245), with participants’ written informed consent. Histologic grading and typing were performed by gynecologic pathologists. Staging was determined using 1988 criteria from the International Federation of Gynecology and Obstetrics. Details of sample information were described in Additional file 1: Table S6. Tissue specimens and blood were obtained at the time of surgery and stored at -70°C until nucleic acids were extracted. All primary tumors evaluated had ≥ 70% neoplastic cellularity. Normal endometrium cells were collected from 10 healthy donors in routine gynecological examinations and were pooled together.

### Library construction, sequencing and mapping

Genomic DNA from tumor tissues and normal endometrium was extracted using the DNeasy Tissue kit (Qiagen, Valencia, CA). MeDIP and MRE sequencing libraries were constructed as previously described [22]. Sequencing reads were aligned to hg19 with Bowtie [107]. MRE reads were normalized to account for differences in enzyme efficiency and scoring consisted of tabulating reads with CpGs at each fragment end [22].

MeDIP-seq data and MRE-seq data of normal tissues, including H1 ESC, PBMC, breast myoepithelial, and fetal brain, were obtained from previous work [19]. This information was listed in Additional file 1: Table S7.

### Affymetrix SNP array 6.0 processing

Affymetrix Genome-Wide SNP Array 6.0 was used for detection of copy number changes in this study. Genomic DNA was extracted from endometrial cancer samples and matched peripheral blood mononuclear cells (PBMC) using the DNeasy Tissue kit (Qiagen, Valencia, CA). The Affymetrix Genome-Wide SNP Array 6.0 standard protocol was performed as recommended by the manufacturer. 500 ng of total genomic DNA were analyzed in the Genome Technology Access Center at Washington University in St. Louis. Data were analyzed by Genotyping Console™ (GTC) Software 4.1.1 following the manufacturer’s instructions.

### RNA isolation

RNA was extracted from frozen endometrial cancer tissues and pooled normal endometrium using Trizol reagent (Life Technologies) following the manufacturer’s instructions with an additional phenol/chloroform extraction to exclude protein contamination. Trace DNA was removed by treatment with TURBO DNA-free Kit (Life Technologies). Quantity and quality of isolated RNA was measured and evaluated by UV spectrophotometer and gel electrophoresis.

### Quantitative real time PCR (qRT-PCR) analysis

The qRT-PCR analyses were performed using the SuperScript VILO cDNA Synthesis Kit (Life Technologies) with iTaq Universal SYBR Green Supermix (Bio-Rad). 500 ng total RNA was used in a 20 ul reverse transcription reaction. The cDNA obtained was diluted to a total volume of 100 ul and stored at -20°C. The primers for human *DNMT1*, *DNMT3A*, *DNMT3B* [108], *DNMT2* [109], *XIST* [110] and candidate human housekeeping gene 18S rRNA [111] were used for amplification of the target genes in normal endometrium and endometrial cancer tissues. All primers were synthesized by Integrated DNA Technologies. The qRT-PCR was performed in a 20 ul reaction consisting of 2 ul diluted cDNA, 0.2 uM of each primer and 10 ul iTaq Universal SYBR Green Supermix. All amplifications were carried out in a Bio-Rad CFX96 Real-Time PCR Detection (Bio-Rad) with denaturation at 95°C for 30 s, followed by 40 cycles at 95°C for 5 s and 60°C for 30 s. A melting curve analysis was performed for each run to confirm the specificity of amplification and lack of primer dimers. The qRT-PCR experiments were always run in triplicate. The relative mRNA expression levels of target genes were quantified using the 2-ΔΔCT equation for endometrial normal and cancer tissues. Mean CT of normal endometrium was used as the calibrator sample.

### DMR identification

The methylMnM package (http://epigenome.wustl.edu/MnM/) was utilized to identify differentially methylated regions (DMRs) in the R 2.15 environment between the DNA methylome of normal endometrium and the DNA methylome of each cancer sample. Default parameters were used, and a statistical cutoff of q-value < 1e-5 was applied to select DMRs from each pair-wise comparison at a resolution of 500 bp. EAC or UPSC DMRs were defined such that the same genomic region must have been called a DMR and have the same direction of DNA methylation change in at least two out of the three cancer vs. normal pairwise comparisons. Endometrial cancer shared DMRs (EC-shared DMRs) were defined as DMRs that were both EAC DMRs and UPSC DMRs and had same direction of DNA methylation change in both types of cancers compared to the pooled normal sample. EAC type-preferred DMRs (tpDMRs) were defined as DMRs in all three EAC samples but not in any UPSC sample; UPSC tpDMRs were defined as DMRs in all three UPSC samples but not in any EAC sample. The same procedure and statistical cutoff were used to define DMRs between H1 ESC and normal endometrium.

### Genomic features

RepeatMasker annotations, CpG islands, and RefSeq Gene coding loci features were all downloaded from the UCSC Genome Browser [112, 113]. 1 KB core promoters were defined as 1 kb around the most 5’ transcription start site (500 bp upstream and 500 bp downstream of TSS) of any RefSeq gene record. microRNA loci were downloaded from mirBASE. The microRNA gene cluster TSSs were download from mirStart (http://mirstart.mbc.nctu.edu.tw/). lincRNA loci were download from the Human lincRNA Catalog (http://www.broadinstitute.org/genome_bio/human_lincrnas/).

### TCGA DNA methylation data

Processed DNA methylation data of uterine corpus endometrial carcinomas (Infinium HumanMethylation450 BeadChip platform) was downloaded from The Cancer Genome Atlas (TCGA) (http://cancergenome.nih.gov/). Quantile normalization was performed across all samples. The beta-value of each probe within DMRs was isolated for further analysis in the R 2.15 environment. Sample histology information was obtained from the supplementary materials of Kandoth et al. [18]. Sample details were described in Additional file 1: Table S8.

### TCGA RNA expression data

Processed mRNA-seq and miRNA-seq data (normalized read counts for each gene, analysis version 2) of uterine corpus endometrial carcinomas (Illumina GA and Illumina HiSeq platform) were downloaded from The Cancer Genome Atlas (TCGA) (http://cancergenome.nih.gov/). RPKM (Reads per kilobase per million) values were computed for each gene using TCGA’s mRNA sequencing data (analysis version 2). The lengths of transcripts were obtained from the NCBI gene bank. Quantile normalization was performed across all samples. RPM (Reads per million) values for each microRNA gene were computed using TCGA’s microRNA sequencing data. All analyses were performed in the R environment (Version 2.15). Sample details were provided in Additional file 1: Table S8.

### Validation of DMRs using TCGA Infinium 450K data

Based on TCGA Infinium 450K data, for any DMR identified in our study that overlaped at least one Infinium probe, we computed the average methylation level (*aML*) in each cancer type group (described in Additional file 1: Table S8) using the beta value of overlapping Infinium probes. Specifically:


where *n* is the number of samples in a specific cancer type group, *w* is the number of available Infinium CpG probes within the corresponding DMR, and *BVij* is beta-value of the *j* th Infinium CpG probe within that DMR in the *i th* sample.

DNA methylation change (*DMC*) were calculated as:


where *aML*_*c*_ is the averaged methylation level of a DMR in a specific cancer type group; *aML*_*n*_ is the averaged methylation level of a DMR in the normal control group.

Based on TCGA Infinium 450K data, all DMRs belonging to EC-shared DMRs,

EAC tpDMRs, and UPSC tpDMRs, were validated by performing the Man-Whitney *U* test to examine the significance of DNA methylation difference between cancer group and normal controls. P-values were corrected for multiple testing by the *Benjamini-Hochberg* method. Validated DMRs were defined by the criteria: 1) the DMR has an adjusted p-value of < 0.05 between the cancer group and normal controls; 2) the DMR has an averaged DNA methylation change of over 0.05 in the cancer group compared to normal controls; 3) the direction of DNA methylation change between cancer and control samples in TCGA Infinium 450K data must fit our predication.

### ENCODE chromHMM chromatin state annotation

ChromHMM annotation of nine ENCODE cell lines [66] were obtained from the UCSC Genome Browser [114]. The nine cell lines are: H1 ESC, GM12878, K562, HepG2, HUVEC, HMEC, HSMM, NHEK, and NHLF. The genome was split to 500 bp windows. For each 500 bp window, we assigned overlapping annotations of “promoter”, “enhancer”, and “insulator” states from these chromHMM maps. In each cell line, the state of each 500 bp window was assigned to the dominant chromHMM state, defined as the state that occupied over 50% of a given window. After merging the data from 9 cell lines, “promoter” regions were defined as the windows have a dominant “promoter” chromHMM state in at least one cell line. “Enhancer” regions were defined as windows have a dominant “enhancer” chromHMM state in at least one cell line but no “promoter” chromHMM state in any cell line.

### ENCODE TFBS and DHS data

ENCODE TFBS data and Dnase I Hypersensitive sites (DHS) data were downloaded from the UCSC Genome Browser ENCODE data portal [114]. TFBS data contained 4,380,444 regions; DHS data contained 1,281,988 regions.

### Enrichment calculation

The binding site enrichment score (*ES*) for each genomic feature, DHS, and transcription factor with respect to DMRs was calculated as:


Where *n* _ *hit* is the number of DMRs that contain specific a genomic feature, experimentally annotated DHS, or TFBS; *n* _ *DMR* is the total number of DMRs; *N* _ *hit* is the number of genomic windows with a specific genomic feature, annotated DHS, or TFBS; *N* _ *all* is the number of 500 bp windows in the human genome (hg19).

### Gene ontology enrichment analysis

Gene Ontology (GO) analyses for biological processes were performed using the GREAT package [115]. Gene regulatory domains were defined by default as regions spanning 5 kb upstream to 1 kb downstream of the TSS (regardless of other nearby genes). Gene regulatory domains were extended in both directions to the nearest gene's basal domain but no more than a maximum extension in one direction. Only categories that were below a false discovery rate of 0.05 were reported.

### Reporter gene assay

Candidate transposable element sequences were amplified from genomic DNA using Pfu-polymerase (Agilent) and primers containing KpnI- or BglII- restriction sites. PCR products were gel-purified using the Qiagen Gel purification kit and then digested by the corresponding restriction enzymes (NEB). The digested PCR products were cloned into the pGL4.23[luc2/minP]-vector (Promega, E8411) using T4-ligase (NEB) and transformed into chemically competent DH5α-cells. The positive clones were verified by enzyme digestion and sequencing. 800 ng of reporter plasmid (or empty pGL4.23[luc2/minP]-vector control) was cotransfected into HEK-293T cell line with the CMV promoter driven *Renilla* luciferase plasmid (200 ng). The luciferase activity was measured 48 hours post transfection and normalized by the relative *Renilla* control. Genomic locations of candidates and primer information were summarized in Additional file 1: Table S9.

### Availability of supporting data

The data set supporting the results of this article are available at the accompanying website (http://epigenome.wustl.edu/Cancer_Epigenome/). Sequencing data has been deposited in the NCBI’s Gene Expression Omnibus repository under the accession number GSE51565.

## Electronic supplementary material

Additional file 1: **Supplementary tables and figures legend.** (DOCX 161 KB)

Additional file 2: **Supplementary figures.** (PDF 6 MB)
