# Supplementary material for: Comparative DNA methylome analysis of endometrial carcinoma reveals complex and distinct deregulation of cancer promoters and enhancers
Source: BMC Genomics. 2014 Oct 6;15(1):868. doi: 10.1186/1471-2164-15-868 (PMC4198682; doi:10.1186/1471-2164-15-868)
Supplement: Supplementary file 1 — Additional file 1: Supplementary tables and figures legend. (DOCX 161 KB) [file 12864_2014_6545_MOESM1_ESM.docx]

**Supplementary Tables**

**Supplementary Table S1. Sequencing data summary**

|  | **MRE-seq** | | | **MeDIP-seq** | | |
| --- | --- | --- | --- | --- | --- | --- |
|  | Total Reads | Filtered Mapped Reads | MRE-CpG site Coverage | Total Reads | Uniquely mapped reads | CpG site Coverage |
| Normal Endometrium | 44749195 | 17642744 | 1676127 | 94754114 | 53448657 | 22297893 |
| EAC-1 | 32919529 | 19079346 | 1823141 | 74344418 | 43926292 | 20987320 |
| EAC-2 | 39035816 | 19921252 | 1953014 | 59295198 | 26281277 | 19550017 |
| EAC-3 | 38648599 | 21660588 | 1768586 | 73391057 | 38834959 | 20915228 |
| UPSC-1 | 43153778 | 16098190 | 2053282 | 68709696 | 37242097 | 20919898 |
| UPSC-2 | 25081733 | 13381446 | 1574349 | 56084324 | 25389877 | 18301589 |
| UPSC-3 | 43381833 | 19785238 | 1781965 | 61454319 | 30733025 | 20331377 |

**Supplementary Table S2. Global DNA methylation changes**

**MeDIP-seq RPKM distribution (percentage) of 5kb window in genome**

| **Methylation level #** | **EAC Sample** | | | **UPSC Sample** | | | **Cancer average** | **Normal endometrium** | **Regions changed** |
| --- | --- | --- | --- | --- | --- | --- | --- | --- | --- |
|  | **1** | **2** | **3** | **1** | **2** | **3** |  |  |  |
| **Low-MeDIP (<0.1)** | 44.4% | 43.2% | 36.6% | 44.4% | 37.4% | 40.0% | 41.0% | 36.3% | 4.7% |
| **Mid-MeDIP (0.1~0.95)** | 44.3% | 46.4% | 54.6% | 44.4% | 53.4% | 50.1% | 48.9% | 55.1% | -6.3% |
| **High-MeDIP (>0.95)** | 11.3% | 10.4% | 8.8% | 11.2% | 9.2% | 9.9% | 10.1% | 8.6% | 1.5% |

# DNA methylation level: MeDIP-seq RPKM in 5kb window.

**Supplementary Table S3. Identified DMRs in 6 cancer samples**

|  | EAC-1 | EAC-2 | EAC-3 | Common DMRs^#^ |
| --- | --- | --- | --- | --- |
| Number of DMRs | 50,672 | 26,270 | 31,429 | 27,009 |

|  | UPSC-1 | UPSC -2 | UPSC -3 | Common DMRs^#^ |
| --- | --- | --- | --- | --- |
| Number of DMRs | 23,914 | 44,673 | 16,901 | 15,676 |

# EAC/UPSC common DMRs were defined such that the same genomic region must have been called a DMR in at least two out of the three cancer vs. normal pairwise comparisons with same direction of DNA methylation change.

**Supplementary Table S4. DMRs distribution with respect to different genomic features**

|  | **Total** | **Intergenic** | **CGI** | **Promoter** | **5’UTR** | **Exon** | **Intron** | **3’UTR** |
| --- | --- | --- | --- | --- | --- | --- | --- | --- |
| **EAC hyper-**  **methylated DMR** | 18294 | 7333 | 4610 | 2761 | 726 | 3320 | 7641 | 790 |
| **EAC hypo-**  **methylated DMR** | 8715 | 4623 | 232 | 276 | 59 | 680 | 3412 | 128 |
| **UPSC hyper-**  **methylated DMR** | 6296 | 2524 | 1126 | 546 | 133 | 1070 | 2702 | 317 |
| **UPSC hypo-**  **methylated DMR** | 9380 | 5475 | 288 | 249 | 48 | 644 | 3261 | 95 |
| **EC shared hyper-**  **methylated DMR** | 4597 | 1771 | 973 | 441 | 110 | 857 | 1969 | 243 |
| **EC shared hypo-**  **methylated DMR** | 2009 | 1035 | 105 | 71 | 19 | 169 | 805 | 22 |

**Supplementary Table S5. DMR distribution on different chromosomes**

|  | EAC | | UPSC | |
| --- | --- | --- | --- | --- |
|  | Hypomethylated | Hypermethylated | Hypomethylated | Hypermethylated |
| chr1 | 568 | 1899 | 454 | 725 |
| chr2 | 506 | 1258 | 469 | 508 |
| chr3 | 251 | 822 | 102 | 362 |
| chr4 | 401 | 741 | 213 | 296 |
| chr5 | 344 | 952 | 763 | 391 |
| chr6 | 289 | 941 | 180 | 442 |
| chr7 | 464 | 907 | 574 | 186 |
| chr8 | 503 | 755 | 627 | 180 |
| chr9 | 403 | 878 | 286 | 322 |
| chr10 | 583 | 1077 | 901 | 301 |
| chr11 | 458 | 922 | 310 | 268 |
| chr12 | 362 | 784 | 482 | 212 |
| chr13 | 201 | 427 | 275 | 152 |
| chr14 | 300 | 553 | 218 | 177 |
| chr15 | 196 | 458 | 95 | 111 |
| chr16 | 624 | 834 | 911 | 258 |
| chr17 | 495 | 1056 | 270 | 394 |
| chr18 | 212 | 439 | 389 | 119 |
| chr19 | 540 | 1084 | 607 | 343 |
| chr20 | 450 | 531 | 538 | 251 |
| chr21 | 206 | 272 | 141 | 131 |
| chr22 | 309 | 448 | 368 | 156 |
| chrX | 50 | 256 | 207 | 11 |

**Supplementary Table S6. Information of studied samples**

| Sample | Histology | Grade | Level | %NPC | MLH1 status |
| --- | --- | --- | --- | --- | --- |
| Normal | Pooled endometrioid without cancer |  |  |  |  |
| UPSC-1 | UPSC | 3 | (severe)3 | 70 | No data |
| UPSC-2 | UPSC | 3 | (severe)3 | 70 | No data |
| UPSC-3 | UPSC | 3 | (severe)3 | 85 | Unmethylated |
| EAC-1 | endometrioid adenocarcinoma | 3 | 1 | 90 | Methylated |
| EAC-2 | endometrioid adenocarcinoma | 3 | 1 | 70 | Methylated |
| EAC-3 | endometrioid adenocarcinoma | 3 | 1 | 80 | Methylated |

**Supplementary Table S7, Methylation data used in this study**

**A: MeDIP-seq datasets used in this study­**

| **Tissue type** | **­Sample ID** | **Sample description** | **GEO ID** |
| --- | --- | --- | --- |
| H1 ES cell  (merged) | H1-ESC-B1 | H1 ESC, Batch1 | GSM543016 |
|  | H1-ESC-B2 | H1 ESC, Batch2 | GSM456941 |
| Blood | PBMC-07 | Blood PBMC, TC007 | GSM613911 |
| Breast | Myo-66 | Breast Myo Epi, RM066 | GSM613857 |
| Brain | F-Brain-01 | Fetal Brain, HuFNSC01 | GSM669614 |

**B: MRE-seq datasets used in this study­**

| **Tissue type** | **­Sample ID** | **Sample description** | **GEO ID** |
| --- | --- | --- | --- |
| H1 ES cell  (merged) | H1-ESC-B1 | H1 ESC, Batch1 | GSM428286 |
|  | H1-ESC-B2 | H1 ESC, Batch2 | GSM450236 |
| Blood | PBMC-07 | Blood PBMC, TC007 | GSM613898 |
| Breast | Myo-66 | Breast Myo Epi, RM066 | GSM613834 |
| Brain | F-Brain-01 | Fetal Brain, HuFNSC01 | GSM669604 |

**Supplementary Table S8. Information of TCGA data used in study**

**A. TCGA Infinium 450K data summary**

| Cancer type | Microsatellite state | Grade | Numbers |
| --- | --- | --- | --- |
| Endometrial adenocarcinoma | Microsatellite instability high  (MSI-H) | 1 | 15 |
|  |  | 2 | 28 |
|  |  | 3 | 42 |
|  | Microsatellite stability  (MSS) | 1 | 35 |
|  |  | 2 | 41 |
|  |  | 3 | 41 |
| Uterine papillary serous carcinoma | Microsatellite stability (MSS) | 3 | 32 |
| Normal control | - | - | 26 |

**B. TCGA mRNA-seq data summary**

| Cancer type | Microsatellite state | Grade | Numbers |
| --- | --- | --- | --- |
| Endometrial adenocarcinoma | Microsatellite instability high  (MSI-H) | 1 | 28 |
|  |  | 2 | 38 |
|  |  | 3 | 58 |
|  | Microsatellite stability  (MSS) | 1 | 59 |
|  |  | 2 | 64 |
|  |  | 3 | 48 |
| Uterine papillary serous carcinoma | Microsatellite stability (MSS) | 3 | 45 |
| Normal control | - | - | 30 |

**C. TCGA miRNA-seq data summary**

| Cancer type | Microsatellite state | Grade | Numbers |
| --- | --- | --- | --- |
| Endometrial adenocarcinoma | Microsatellite instability high (MSI-H) | 3 | 58 |
|  | Microsatellite stability (MSS) | 3 | 50 |
| Uterine papillary serous carcinoma | Microsatellite stability (MSS) | 3 | 42 |
| Normal control | - | - | 22 |

**Supplementary Table S9. Primers used in reporter assay of this study**

| **Location** | **Primer-F** | **Primer-R** | **Length** | **Target** |
| --- | --- | --- | --- | --- |
| chr6:155534337-155535395 | ATCGGCTCGAGAGGAAGAAGGAGGTATGCGG | CGTTCAAGCTTCAGTCACTGCGATGATGCC | 1059bp | MER52A |

**Supplementary Figures**

**Supplementary Figure S1.**

**(A).** Genome-wide MeDIP-seq RPKM distribution of 5kb windows in 7 samples. Values greater than 1 were trimmed to 1. Green line: normal endometrium. Blue dashed line: 3 EAC samples. Red dash line: 3 UPSC samples. Red box: shift of MeDIP-seq RPKM distribution in tumors.

**(B).** *DNMT1*, *DNMT2*, *DMNT3A*, and *DNMT3B* abundance quantified by qRT-PCR in all 7 samples with 3 technical replicates. Fold expression +/- S.E. relative to normal endometrium.

**(C).** Gene expression of *DNMT1*, *DMNT3A*, and *DNMT3B* in normal controls and pre-classified (grades, microsatellite stability, subtype) endometrial cancers. Y-axis: RPKM value based on mRNA-seq from TCGA. MSI-H: Microsatellite instability high. MSS: Microsatellite stability.

**(D).** *XIST* abundance quantified by qRT-PCR in all 7 samples by 3 technical replicates. Fold expression +/- S.E. relative to normal endometrium.

**Supplementary Figure S2.**

**(A).** Open chromatin feature enrichment for EAC DMRs and UPSC DMRs. **Left**: percentage of DMRs that overlapped ENCODE DHS and TFBS annotations. **Right**: enrichment of DMRs that overlapped ENCODE DHS and TFBS annotations.

**(B).** Distribution (percentage) of endometrial cancer hypermethylated DMRs (left) and hypomethylated DMRs (right) in different genomic features.

**(C).** Percentage of DRMs containing Infinium probes in EC-shared DMRs, EAC tpDMRs, and UPSC tpDMRs.

**(D).** Percentage of validated EC-shared DMRs, EAC tpDMRs, and UPSC tpDMRs in grade 3 MSI-H type EAC and grade 3 MSS type UPSC cancer samples.

**(E).** Hierarchical clustering of 7 samples based on DNA methylation level (represented by MeDIP-seq data) of effected CpG islands. Values greater than 8 were trimmed to 8.

**Supplementary Figure S3.**

**(A).** Gene function enrichment analysis of EAC tpDMRs and UPSC tpDMRs by GREAT tool. X-axis denotes negative log10 transformed p-value.

**(B).** Gene function enrichment analysis of RefSeq genes with DMRs in 1kb core promoter by DAVID tool. X-axis denotes negative log10 transformed p-value.

**Supplementary Figure S4.**

**Top:** Gene expression analysis of tumor suppressor genes with hypermethylated promoters in normal controls and grade 3 pre-classified (microsatellite stability, subtype) endometrial cancers. Genes with significant changes in expression were shown. Y-axis: RPKM value based on mRNA-seq from TCGA. Caret indicates P <0.05, asterisk indicates P <0.01, octothorpe indicates P <1e-5, Student's t-Test.

**Bottom:** Epigenome Browser views of 22 tumor suppressor gene promoters with increased DNA methylation across cancer samples. MeDIP-seq tracks were displayed. The gene set view (-2.5kb to +2.5kb regions around TSS) was made by the WashU Epigenome Browser.

**Supplementary Figure S5.**

**(A):** Gene expression analysis of tumor suppressor genes with hypermethylated promoters in normal controls and grade 3 pre-classified (microsatellite stability, subtype) endometrial cancers. Genes with significant changes in expression were shown. Y-axis: RPKM value based on mRNA-seq from TCGA. Caret indicates P <0.05, asterisk indicates P <0.01, octothorpe indicates P <1e-5, Student's t-Test.

**(B):** Epigenome Browser views of 21 tumor suppressor gene promoters with increased DNA methylation across cancer samples. MeDIP-seq tracks were displayed. The gene set view (-2.5kb to +2.5kb regions around TSS) was made by the WashU Epigenome Browser.

**(C):** Methylation level of *MLH1* promoter in normal controls and grade 3 pre-classified (microsatellite stability, subtype) endometrial cancers. Each boxplot represents the distribution of averaged methylation levels of CpG probes located in the *MLH1* promoter in cancer groups and normal controls (TCGA Infinium 450K data).

**Supplementary Figure S6.**

**Hypomethylation in promoters of tumor suppressor genes *CDH1* and *SFN*.**

**(A)** Epigenome Browser views of two gene promoters with decreased DNA methylation in three endometrioid adenocarcinoma samples. MeDIP-seq tracks were displayed. The gene set view (-3kb to +3kb regions around TSS) was made by the WashU Epigenome Browser.

**(B)** Gene expression analysis of tumor suppressor genes with hypermethylated promoters in normal controls and grade 3 pre-classified (microsatellite stability, subtype) endometrial cancers. Y-axis: RPKM value based on mRNA-seq from TCGA. Caret indicates P <0.05, asterisk indicates P <0.01, octothorpe indicates P <1e-5, Student's t-Test.

**(C)** Methylation level of 10 CpG sites in the promoter of *CDH1* in grade 3 MSI-H type EAC and normal controls (TCGA Infinium 450K data). Two CpGs (cg17655614 and cg11667754) located in a DMR show significant demethylation in EAC. Another 8 CpGs located in the *CDH1* promoter CpG island did not show methylation change. Mann–Whitney U test was performed for each CpG probe between EAC and normal controls.

**Supplementary Figure S7.**

**Global DNA methylation change on chromosome 10.** MeDIP-seq and MRE-seq RPKM values of 7 samples were calculated at 500kb resolution across the chromosome 10. The averaged RPKM fold-changes (cancer/normal) of each type (3 EACs and 3 UPSCs) were log2-transformed and plotted along with coordinate of chromosome 10.

**Supplementary Figure S8.**

**(A)**. Numbers of miRNA clusters and lncRNA with hypermethylated or hypomethylated promoters in EAC and UPSC.

**(B)**. Gene expression analysis of miRNA with hypermethylated promoter in normal controls and grade 3 pre-classified (microsatellite stability, subtype) endometrial cancers. Genes with significant changed expression were shown. Y-axis: RPM value based on smRNA-seq from TCGA. Caret indicates P <0.05, asterisk indicates P <0.01, octothorpe indicates P <1e-5, Student's t-Test.

**(C)**. Gene expression analysis of miRNA with hypomethylated promoters in normal controls and grade 3 pre-classified (microsatellite stability, subtype) endometrial cancers. Genes with significant changes in expression were shown. Y-axis: RPM value based on smRNA-seq from TCGA. Caret indicates P <0.05, asterisk indicates P <0.01, octothorpe indicates P <1e-5, Student's t-Test.

**(D)**. Epigenome Browser views of *MIR200B-MIR200A-MIR429* cluster with decreased DNA methylation in endometrial cancers. MeDIP-seq tracks were displayed.

**(E)**. Significantly upregulated miRNAs with hypermethylated promoters in normal controls and grade 3 pre-classified (microsatellite stability, subtype) endometrial cancers. Y-axis: RPM value based on smRNA-seq from TCGA. Caret indicates P <0.05, asterisk indicates P <0.01, octothorpe indicates P <1e-5, Student's t-Test.

**Supplementary Figure S9.**

**(A)**. Epigenome Browser views of lncRNA *MEG3* promoter with increased DNA methylation in endometrial cancers. MeDIP-seq tracks were displayed.

**(B)**. Gene expression of *MEG3* in normal controls and grade 3 pre-classified (microsatellite stability, subtype) endometrial cancers. Y-axis: RPKM value based on mRNA-seq from TCGA. Student's t-Test.

**Supplementary Figure S10.**

**(A).** Functional enrichment by GREAT analysis of DMRs classified by the patterns ***MMU***, ***UUM***, and ***MUM***. X-axis denotes negative log10 transformed p-value.

**(B).** Gene expression of *ADCY3* in normal controls and grade 3 pre-classified (microsatellite stability, subtype) endometrial cancers. Y-axis: RPKM value based on mRNA-seq from TCGA. Student's t-Test.

**Supplementary Figure S11.**

**DNA methylation change of transposable element families.** The MeDIP-seq and MRE-seq RPKM fold-changes (cancer/normal) of each type (3 EACs and 3 UPSCs) were normalized by RPKM value in normal endometrium.

MeDIP-seq and MRE-seq RPKM were calculated as:

RPKM= (r_te *1e9) / (r_total * len_te)

r_te is total unique reads mapped to all copies of the same transposable element family. r_total is all unique reads mapped to genome. len_te is total length of all copies of the same transposable element family.

**Supplementary Figure S12.**

**Methylation changes of all copies of the *LTR6A* and *MER52A* subfamilies across 7 samples.** MeDIP-seq and MRE-seq RPKM of individual *LTR6A* and *MER52A* copies were calculated in 3 EACs and 3 UPSCs, respectively. RPKM values were subtracted by values from normal endometrium sample before clustering.
