# Supplementary material for: Comparative DNA methylome analysis of endometrial carcinoma reveals complex and distinct deregulation of cancer promoters and enhancers
Source: BMC Genomics. 2014 Oct 6;15(1):868. doi: 10.1186/1471-2164-15-868 (PMC4198682; doi:10.1186/1471-2164-15-868)

Supplementary Fig.S1

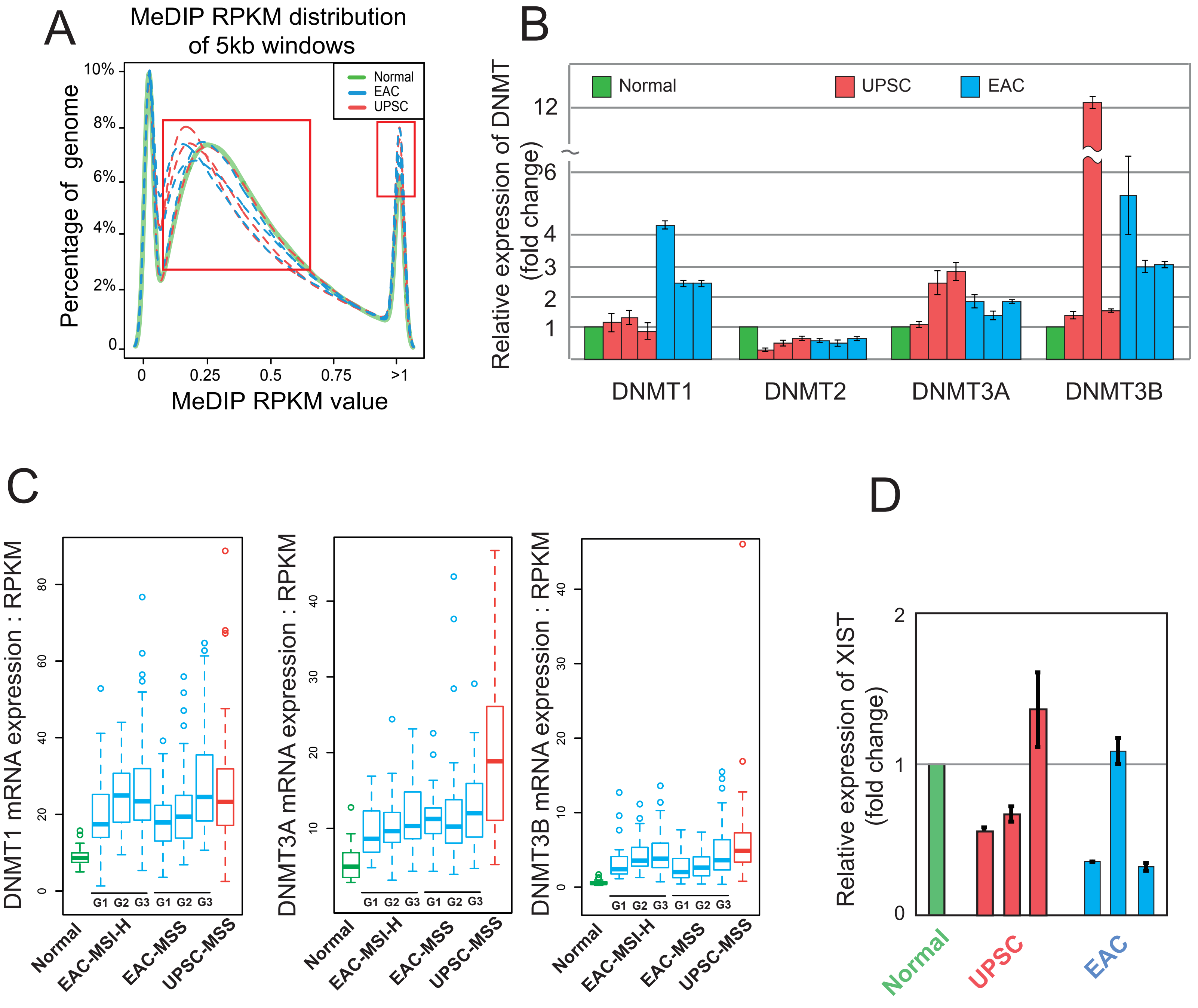

Supplementary Fig.S2

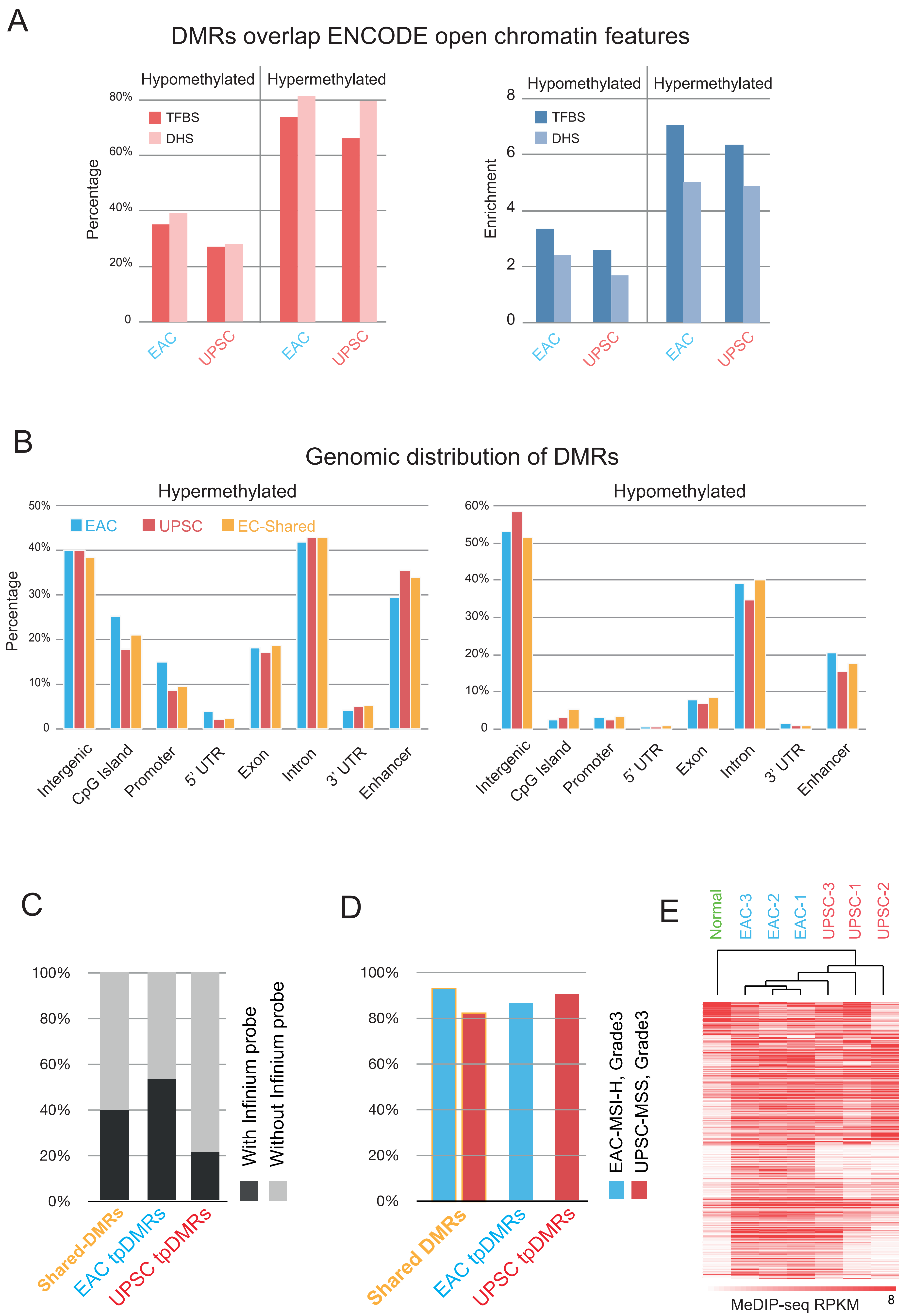

Supplementary Fig.S3

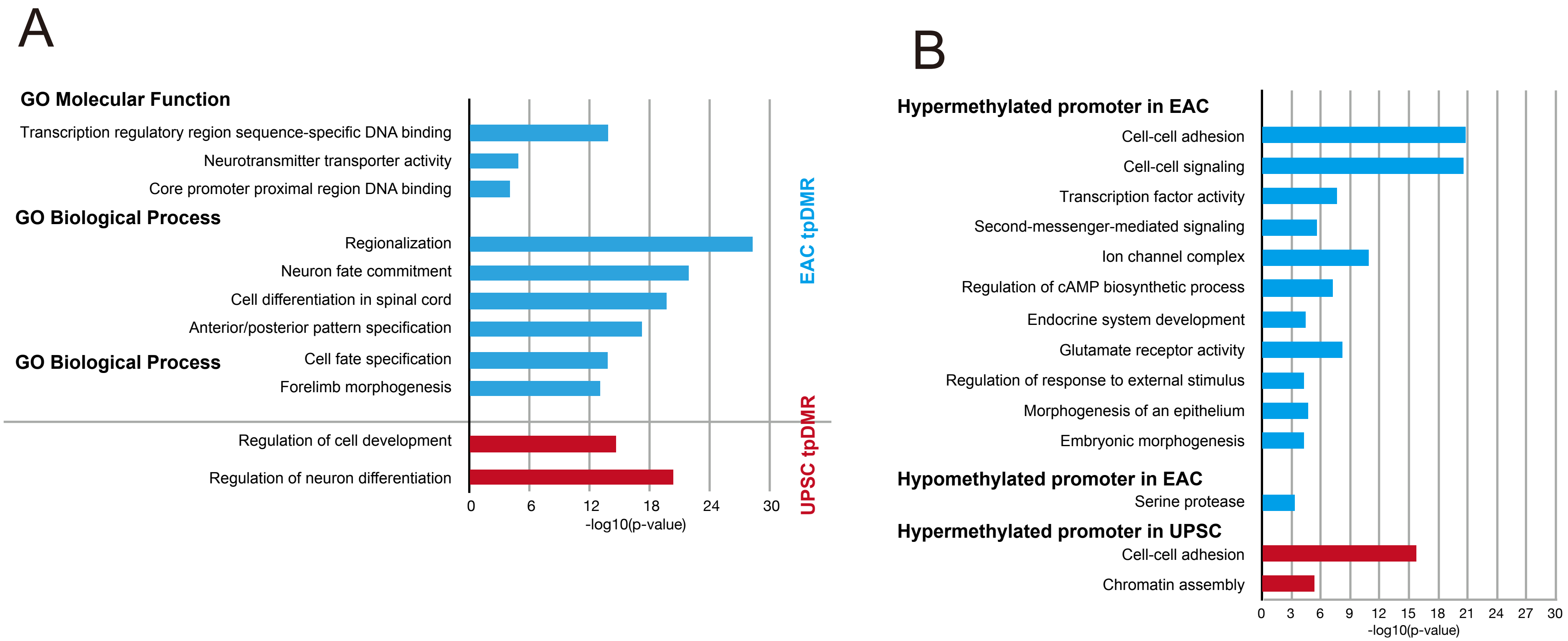

Supplementary Fig.S4

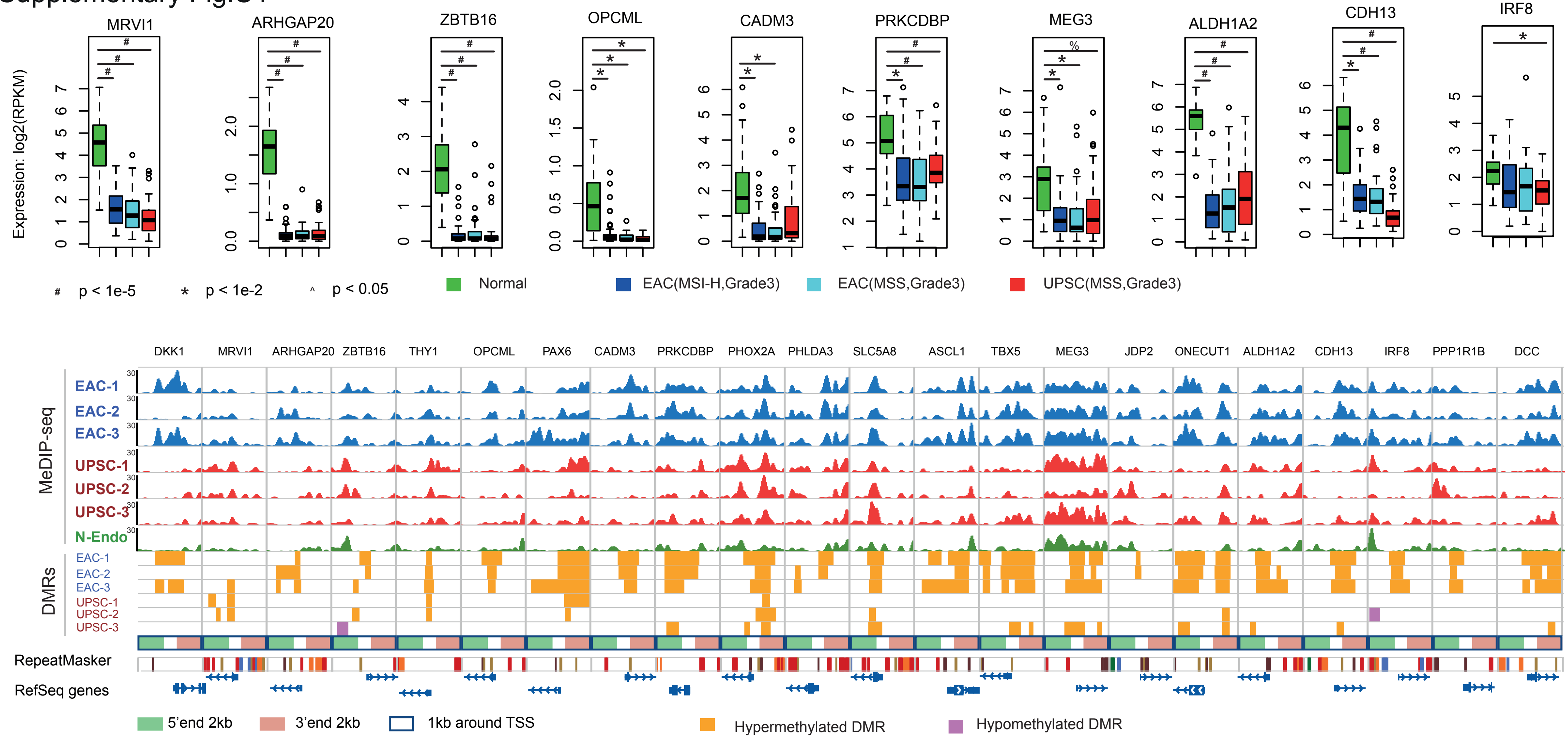

Supplementary Fig.S5

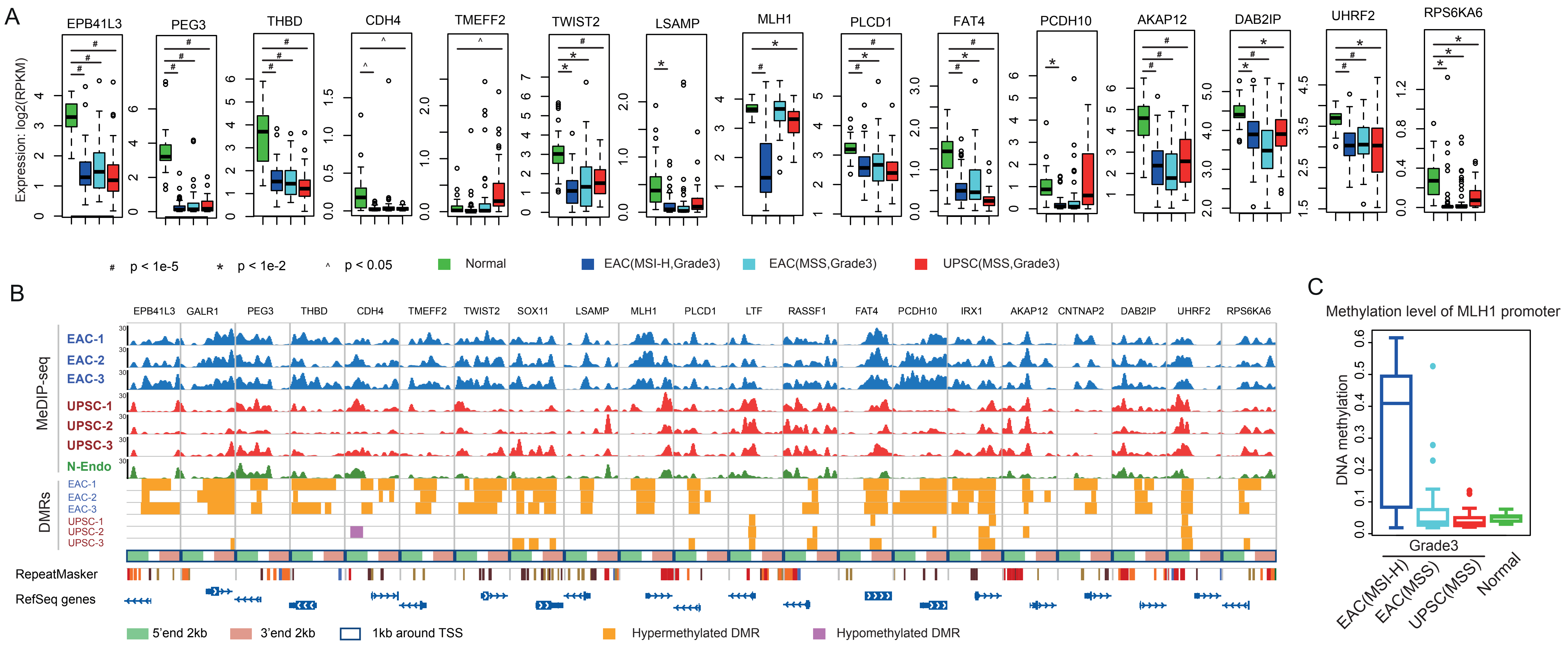

Supplementary Fig.S6

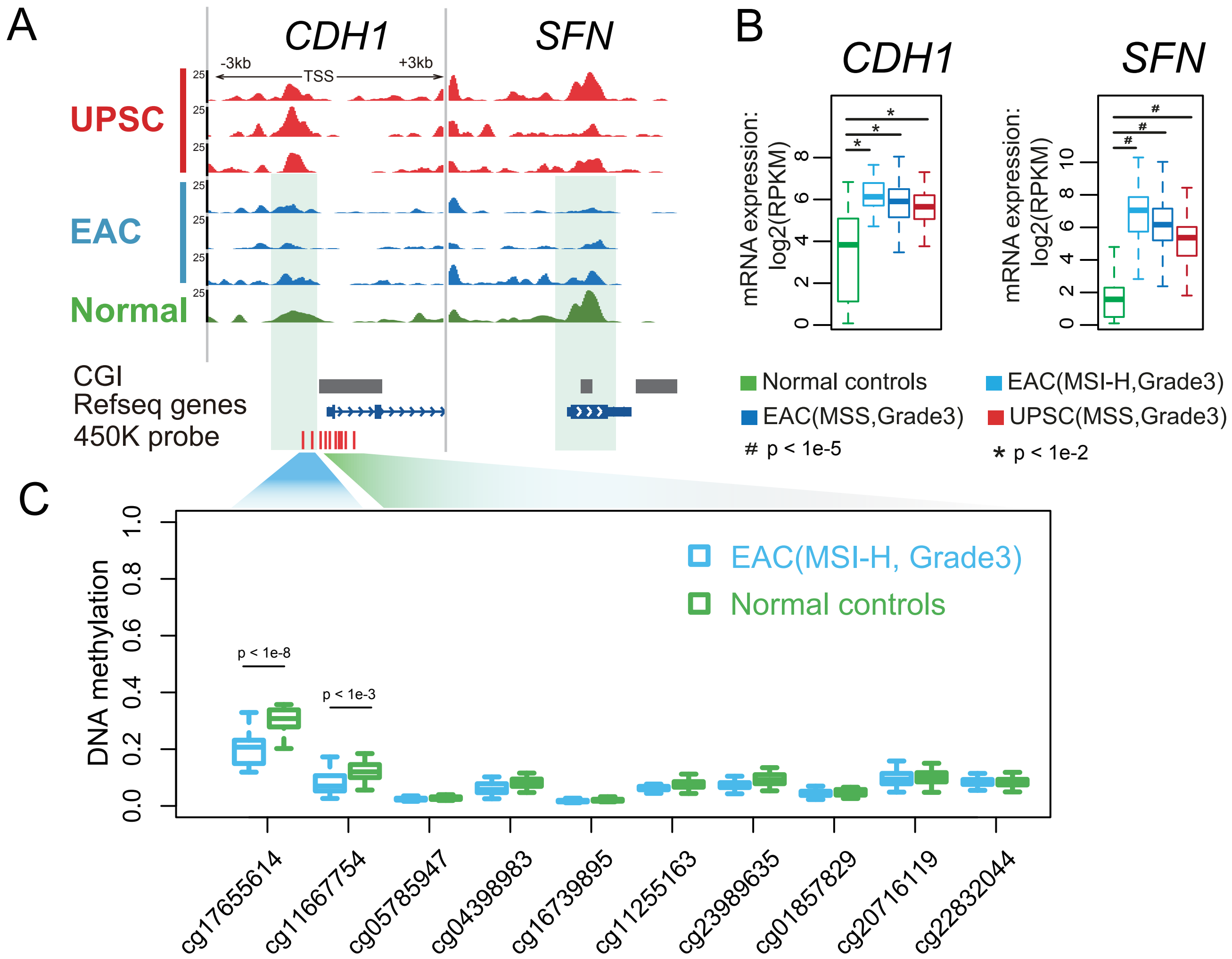

Supplementary Fig.S7

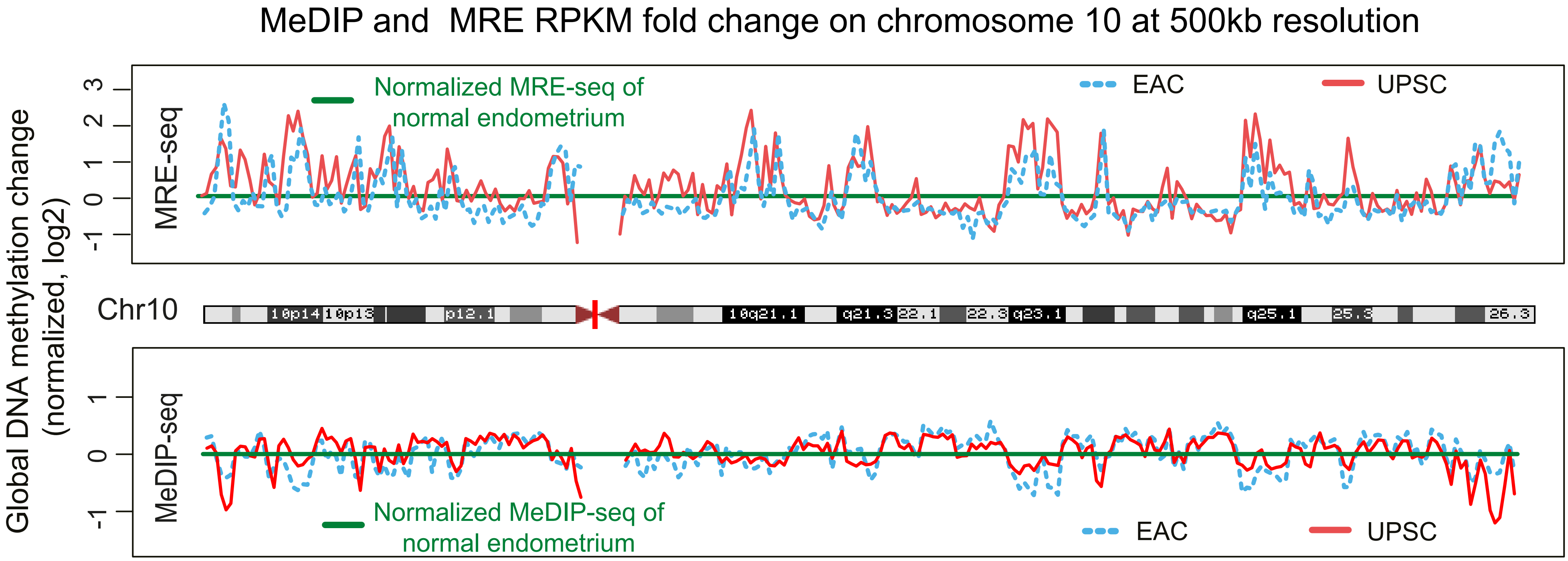

Supplementary Fig.S8

A

		EAC	UPSC
**MiRNA-cluster**	Hypermethylated	24	9
	Hypomethyalted	6	2
**lncRNA**	Hypermethylated	621	168
	Hypomethyalted	207	245

B

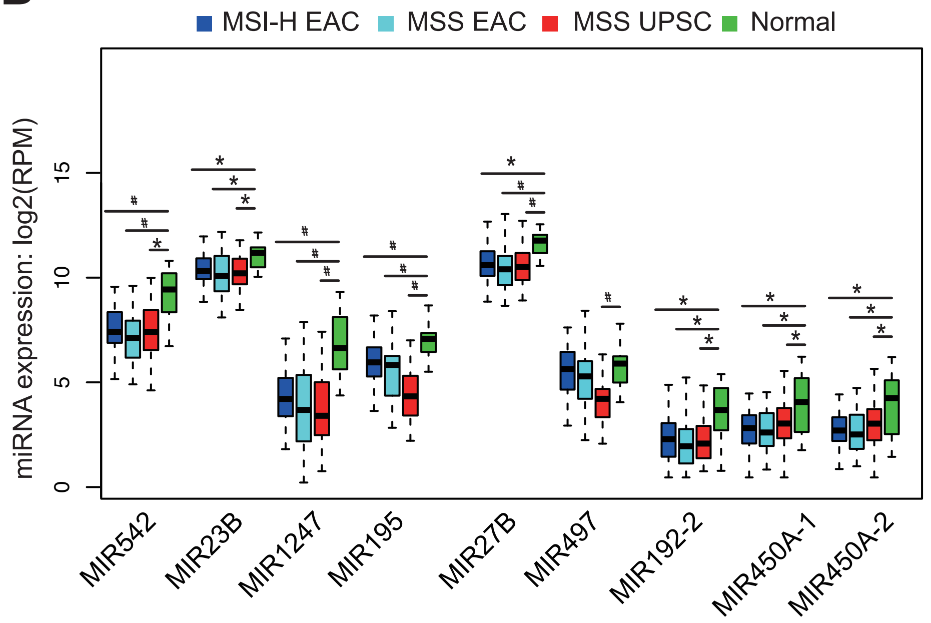

C

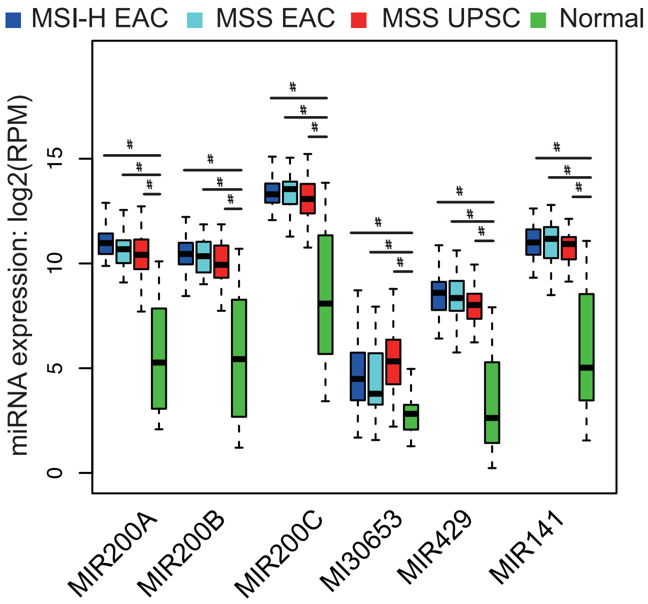

E

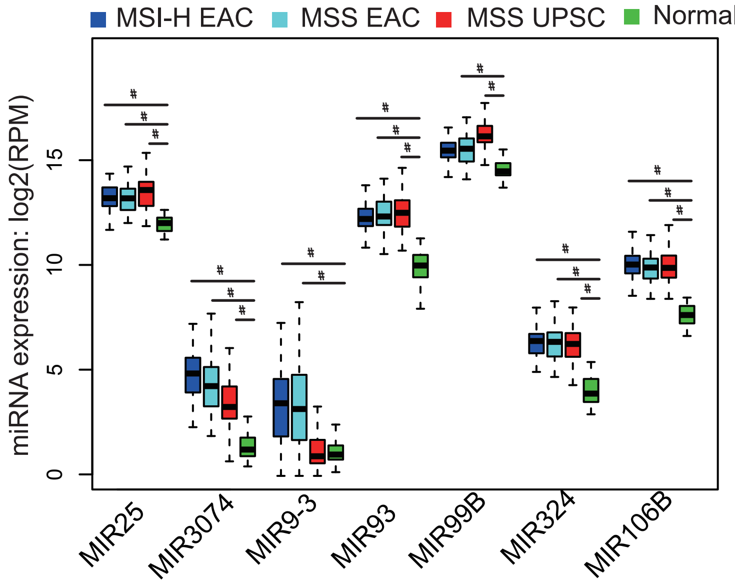

D

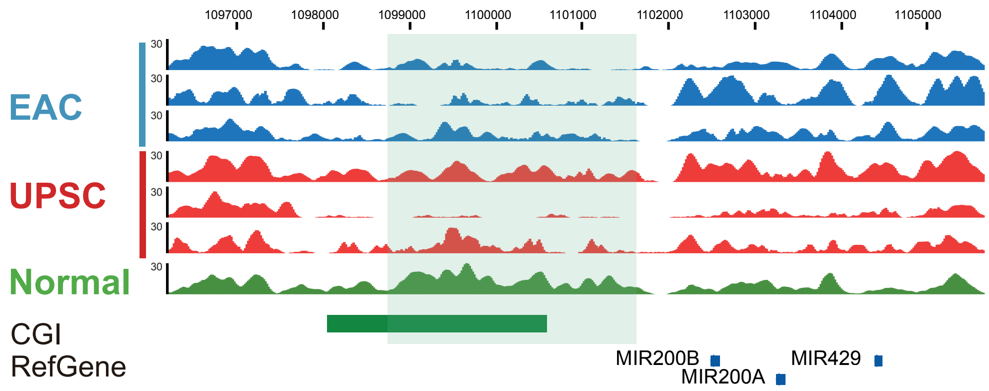

Supplementary Fig.S9

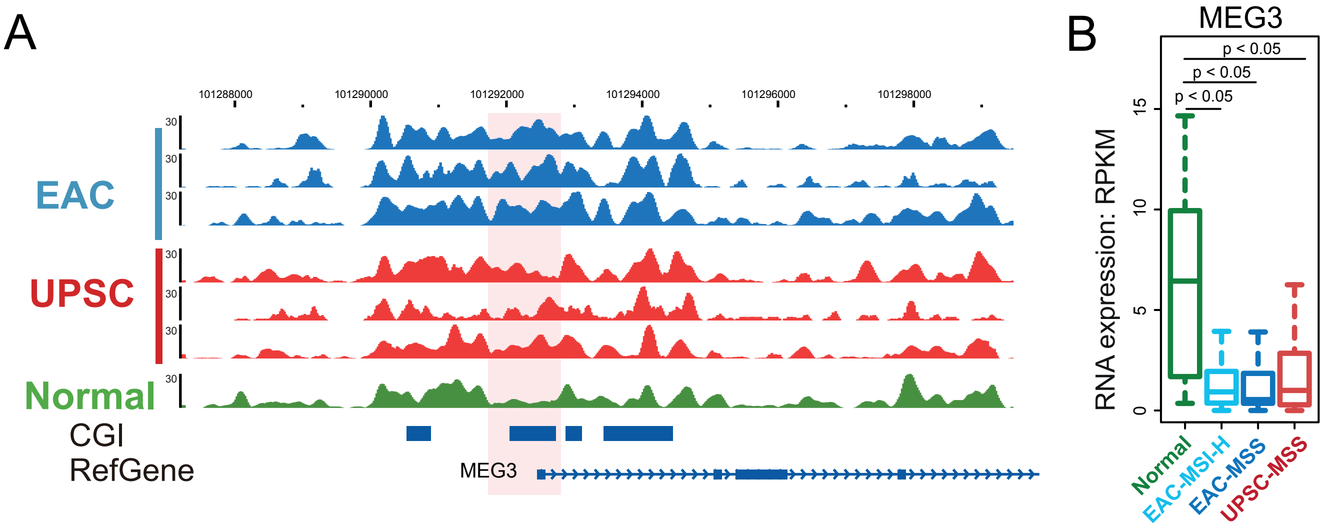

Supplementary Fig.S10

A

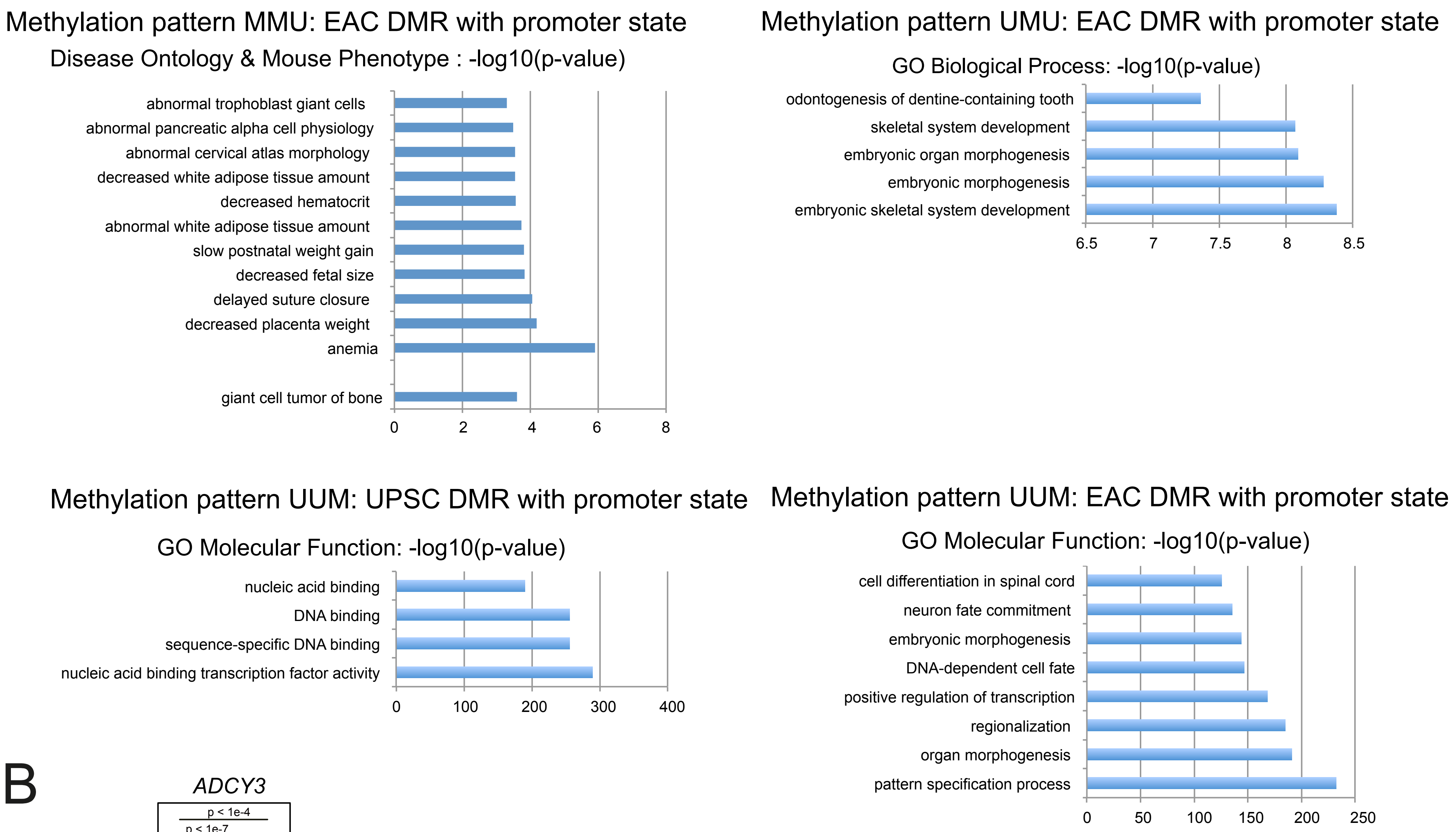

B

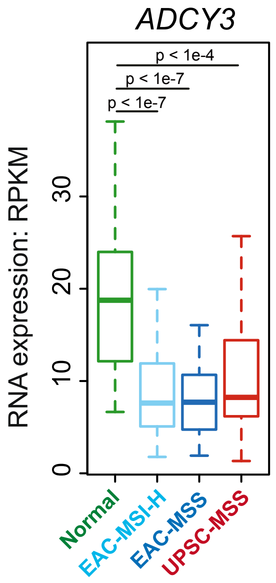

Supplementary Fig.S11

DNA methylation change of transposable element families

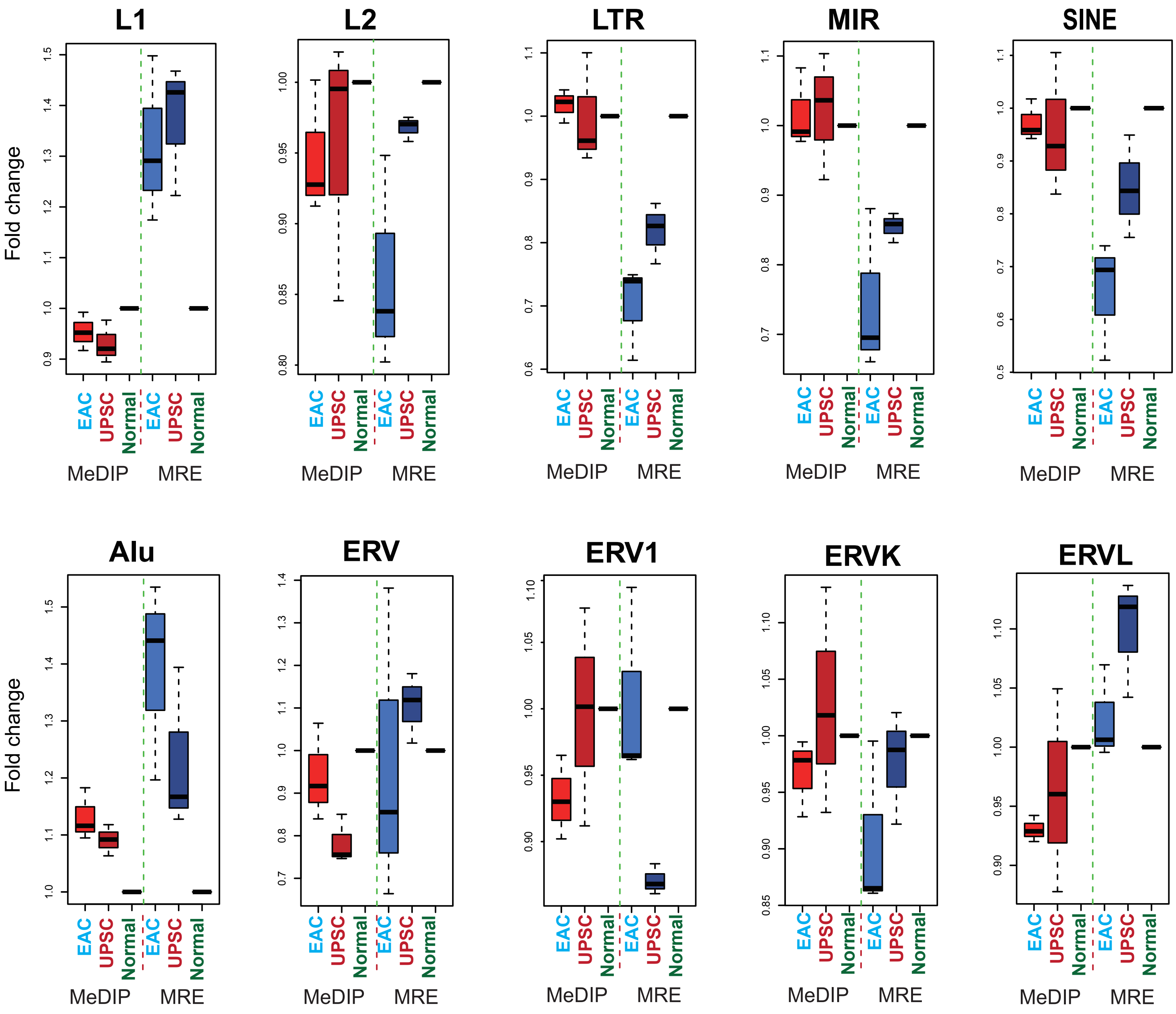

Supplementary Fig.S12

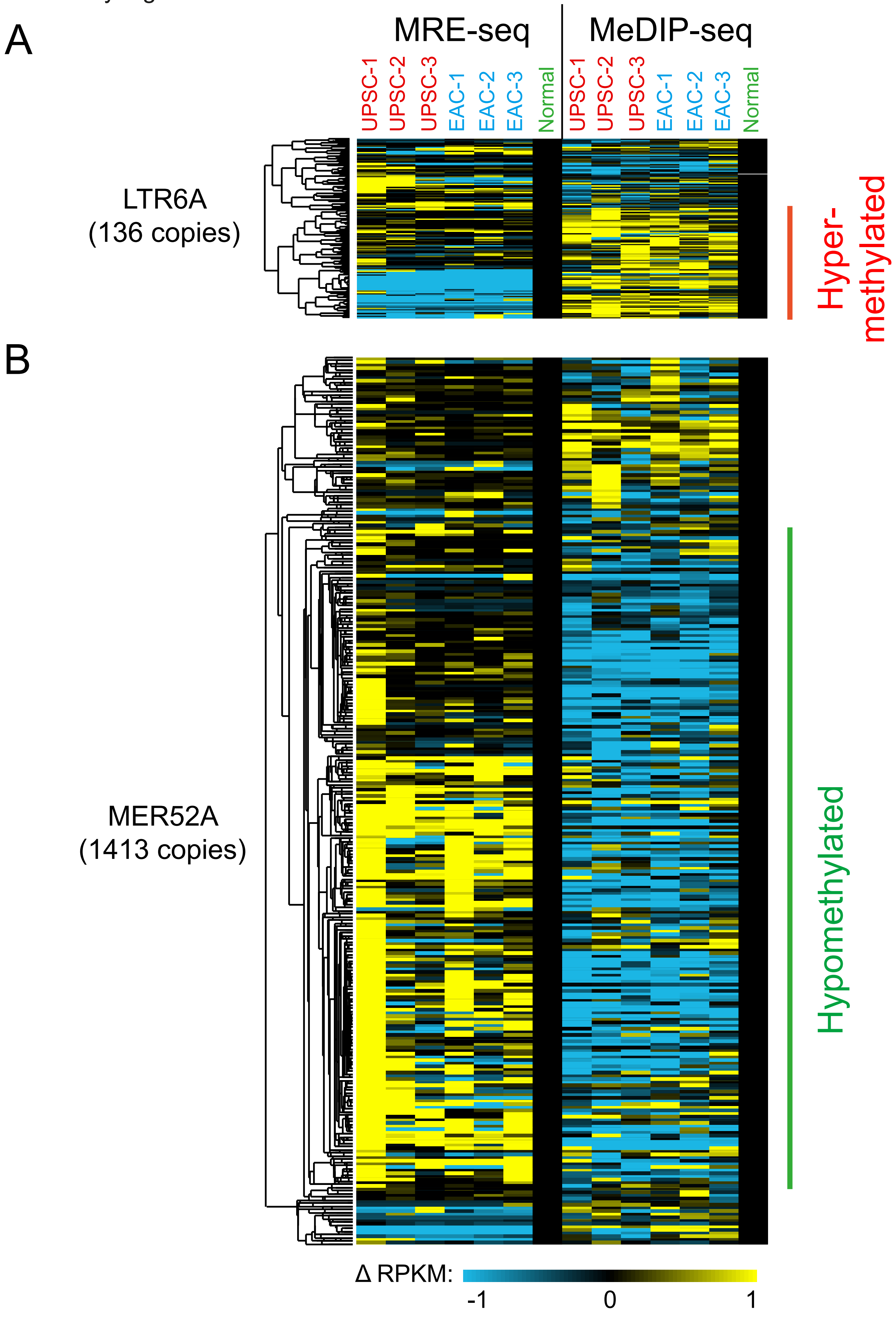

Supplement: Supplementary file 2 — Additional file 2: Supplementary figures. (PDF 6 MB) [file 12864_2014_6545_MOESM2_ESM.pdf]
